# Natural Compounds and Their Microbiota-Derived Metabolites as Modulators of the Microbiota–Gut–Brain Axis in Alzheimer Disease: Biochemical Mechanisms and Drug Discovery Insights—A Narrative Review

**DOI:** 10.3390/molecules31203728

**Published:** 2026-10-17

**Authors:** Maria Maddalena Cavalluzzi, Francesco Terlizzi, Alessia Catalano, Alessandra Pannunzio, Natalie Paola Rotondo, Solomon Habtemariam, Giovanni Lentini

**Affiliations:** 1 Department of Pharmacy—Pharmaceutical Sciences, University of Bari Aldo Moro, Via Orabona, 4, 70126 Bari, Italy; f.terlizzi8@phd.uniba.it (F.T.); alessia.catalano@uniba.it (A.C.); alessandra.pannunzio@uniba.it (A.P.); natalie.rotondo@uniba.it (N.P.R.); giovanni.lentini@uniba.it (G.L.); 2 Pharmacognosy Research & Herbal Analysis Services UK, 124 City Road, London EC1V 2NX, UK; s.habtemariam@herbalanalysis.co.uk

**Keywords:** neurodegenerative diseases, Alzheimer disease, natural bioactive compounds, polyphenols, microbial metabolites, microbiota–gut–brain axis

## Abstract

The microbiota–gut–brain axis (MGBA) is a complex signaling network linking the gut microbiota with central nervous system function. Growing evidence suggests that gut microbial dysbiosis contributes to neurodegenerative diseases (NDs) through mechanisms involving oxidative stress, neuroinflammation, disruption of the epithelial barrier, and altered microbial metabolite production. Unfortunately, the currently available therapies for NDs remain predominantly focused on symptomatic relief and do not modify the underlying disease progression. Natural bioactive compounds, particularly polyphenols, have emerged as promising multi-target modulators of the MGBA due to their pleiotropic effects across microbial, epithelial, immune, and neural compartments. This narrative review summarizes the available evidence on natural compounds and their microbial metabolites that target the MGBA, as evaluated in both in vitro and in vivo models. With a focus on Alzheimer disease, the molecular and cellular mechanisms underlying their effects across microbial, epithelial, immune, and neural compartments, as well as their potential therapeutic value through MGBA modulation, are discussed.

## 1. Introduction

Neurodegenerative diseases (NDs) represent a heterogeneous group of progressive disorders and include Alzheimer’s disease (AD), Parkinson’s disease (PD), Huntington’s disease (HD), and amyotrophic lateral sclerosis (ALS). They are characterized by gradual and largely irreversible loss of neuronal structures and functions, leading to cognitive, motor, and behavioral impairment. These disorders substantially differ from each other in their clinical manifestations, neuropathological hallmarks, and the specific brain regions and neuronal populations they primarily affect. On the other hand, they share several common mechanisms that contribute to progressive neuronal dysfunction and loss, including oxidative stress, mitochondrial dysfunction, neuroinflammation, innate immune activation, and impaired cellular homeostasis.

According to the World Health Organization (WHO), AD accounts for approximately 60–70% of dementia cases, which affects an estimated 57 million people globally in 2021 [1]. Given that advancing age is a major risk factor for AD and many other neurodegenerative diseases, the rapidly growing global ageing population is further expected to contribute to the increasing burden of these disorders. Hence, understanding the interconnected molecular pathways underlying neurodegeneration is essential for identifying therapeutic targets and accelerating the development of effective disease-modifying therapies [2,3,4,5]. A key pathological event in neurodegeneration is redox imbalance, which arises when the production of reactive oxygen species (ROS) and reactive nitrogen species (RNS) exceeds the capacity of endogenous antioxidant systems to neutralise and eliminate them. Persistent redox imbalance leads to a state of oxidative stress [6], which promotes neuronal cellular damage and ultimately contributes to neuronal dysfunction and death [7]. As a key modulator of neuronal apoptosis, mitochondrial dysfunction, and disease progression, the role of oxidative stress in NDs is now well established. At the cellular level, the vulnerability of neurons to oxidative stress is even more evident given their high metabolic demands and sustained oxygen consumption to support their energy-intensive functions [8]. Moreover, recent evidence suggests that oxidative stress tends to increase with advancing age, as age-related declines in antioxidant defences and mitochondrial function can promote the accumulation of ROS and RNS [9]. Hence, oxidative stress is widely recognized as a key contributor to the pathogenesis of NDs and represents a potential therapeutic target [7].

Another well-established mechanism implicated in neurodegenerative diseases is neuroinflammation, which is closely interconnected with oxidative stress. Oxidative stress can promote the activation of inflammatory signalling pathways and the expression of pro-inflammatory genes, thereby contributing to sustained neuroinflammatory responses. Similarly, activated immune cells can generate ROS and RNS, further exacerbating oxidative stress and establishing a self-perpetuating cycle of inflammation and oxidative damage in neurodegeneration [10].

It has been widely demonstrated that neuroinflammatory responses can enhance neuronal excitability, promote neuronal injury, and increase blood–brain barrier (BBB) permeability, thereby impairing its protective function [11]. Reactive astrocytes and microglia, the principal mediators of innate immunity in the brain, promote the release of inflammatory mediators such as cytokines, chemokines, and secondary messengers, disrupting neuronal homeostasis and contributing to neuronal injury. Therefore, chronic neuroinflammation represents another key hallmark of NDs.

The complexity of neurodegenerative diseases is reflected by the fact that current pharmacological approaches largely focus on symptomatic relief by using relatively few available disease-modifying therapies. For example, pharmacological treatments for AD include cholinesterase inhibitors such as donepezil, rivastigmine, and galantamine, as well as the NMDA receptor antagonist memantine, which primarily target cognitive and functional symptoms. Similarly, dopaminergic therapies used in PD can alleviate motor symptoms without effectively halting or reversing the progressive neurodegeneration underlying the disease [12]. In recent years, growing evidence has highlighted the pivotal role of the gut–brain axis in central nervous system (CNS) regulation. In particular, the microbiota–gut–brain axis (MGBA) is now recognized as a bidirectional signaling system integrating neural, metabolic, immune, and endocrine pathways, thereby influencing brain function and neuroinflammatory status [13]. Conversely, alteration in gut microbial composition, commonly referred to as dysbiosis, has been increasingly reported in neurodegenerative conditions, including AD and PD [14,15]. An in-depth discussion of this topic can be found in previous reviews [13,16,17,18].

Considering the multifactorial nature of NDs and the limitations of single-target therapies, increasing attention has been paid to multi-target strategies addressing oxidative stress, inflammatory pathways, cellular homeostasis, and the MGBA. In this context, given the interplay between diet and gut microbiota, natural bioactive compounds have gained attention as promising therapeutic candidates due to their pleiotropic properties, including modulation of gut microbial composition [19], oxidative stress, neuroinflammatory pathways [20], immune signaling, and intestinal permeability [21,22]. These compounds may therefore contribute to maintaining CNS homeostasis and modulating neuroinflammatory responses.

In this narrative review, we summarize the most relevant and recent studies investigating the effects of natural compounds on MGBA, either in their native form or as their corresponding metabolites generated by the intestinal microbiota. The molecular and cellular mechanisms underlying their neuroprotective activity in NDs are discussed, with special attention given to AD. Although previous reviews have discussed microbiota-derived metabolites in NDs, an integrated overview of their effects on the main biological processes involved in AD remains valuable. Accordingly, this review examines the effects of natural compounds and their microbiota-derived metabolites on the key processes involved in neurodegeneration, including intestinal and blood–brain barrier (BBB) integrity, oxidative stress, neuroinflammation, microglial activation, mitochondrial dysfunction, and autophagy/mitophagy. These processes have also been summarized in simplified schemes to enhance readability and facilitate a broader understanding of the concepts discussed, particularly among readers with a medicinal chemistry background. Although experimental evidence supporting pharmaceutical optimization of microbial metabolites remains limited to date, a medicinal chemistry section is dedicated to this emerging perspective by discussing their potential as starting points for the development of metabolite-inspired compounds possibly endowed with improved drug-likeness.

## 2. Literature Search Strategy

The literature was retrieved from electronic databases, including PubMed and Google Scholar, and supplemented by manual screening of the reference lists of relevant articles. Searches were conducted separately in the two databases using combinations of keywords such as “natural bioactive compounds”, “microbiota”, “MGBA”, “neurodegenerative diseases”, “polyphenols”, and “microbial metabolites”. In PubMed, keywords were combined using Boolean operators (AND/OR); Google Scholar was used for broader keyword-based searches and for identifying additional relevant publications. Article selection was based primarily on relevance to the scope of the review. Particular emphasis was placed on studies published within the last five years (2021–2026), while older publications were retained when considered relevant to the understanding of established biological mechanisms.

Given the narrative nature of the review and the iterative search process, a formal record-counting and screening procedure was not performed. No specific language restriction was applied. The literature search was last updated in September 2026.

## 3. Key Biological Pathways Involved in MGBA Modulation by Natural Compounds

Natural matrices commonly consumed through the diet contain a composition of structurally diverse bioactive compounds that act on multiple molecular targets simultaneously. Hence, they offer a multi-target therapeutic strategy particularly relevant for multifactorial diseases such as NDs. Understanding the key pathways through which natural compounds modulate MGBA function is therefore essential for elucidating their potential role in neuroprotection and disease management. Only the mechanisms involving natural compounds will be considered herein.

### 3.1. Preservation of Intestinal Integrity

In the gut compartment, increased permeability (known as “leaky gut”) is increasingly recognized as a contributor to neurodegeneration [23]. Disruption of tight junction complexes, including occludin, claudins (e.g., claudin-1), and zonula occludens-1 (ZO-1), compromises intestinal barrier integrity, thereby increasing paracellular permeability and facilitating the systemic translocation of endotoxins (so-called endotoxemia), microbial products, and pro-inflammatory mediators into the circulation. This process can trigger peripheral immune activation and contribute to BBB dysfunction [14]. Therefore, preservation of intestinal barrier integrity seems to be closely interconnected with the maintenance of BBB function.

**Figure 1:**
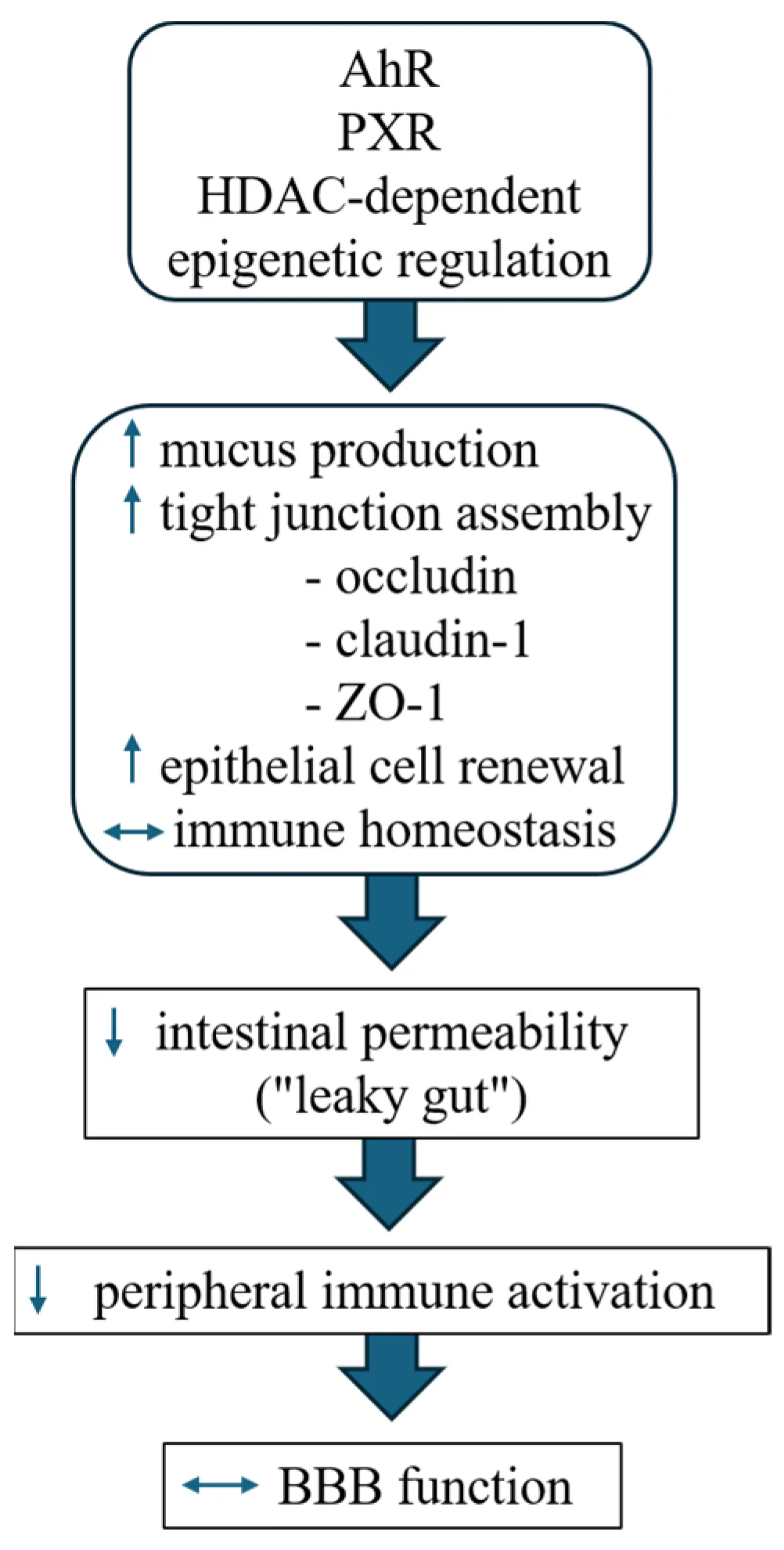
Schematic overview of mechanisms preserving intestinal barrier integrity and gut–brain axis stability: upward arrows (↑) indicate an increase, downward arrows (↓) indicate a decrease, and double-ended arrows indicate preservation of physiological conditions. Abbreviations: AhR, aryl hydrocarbon receptor; BBB, blood–brain barrier; HDAC, histone deacetylase; PXR, pregnane X receptor; ZO-1, zonula occludens-1. Authors’ original figure obtained through Microsoft PowerPoint.

An integrated network of microbiota-targeted bioactive compounds, including polyphenols [24,25], prebiotics [26], and microbial metabolites generated from the biotransformation of phytochemicals [27], plays a crucial role in the preservation of intestinal epithelial integrity through coordinated regulation of barrier function [27,28]. Modulation of mucus production, tight junction assembly, epithelial cell renewal, and immune homeostasis, mediated by aryl hydrocarbon receptor (AhR) and pregnane X receptor (PXR) signaling, as well as histone deacetylase (HDAC)-dependent epigenetic regulation of barrier-related gene expression, may support gut–brain axis stability (see Figure 1).

A detailed discussion of barrier integrity within the gut–brain axis and the role of the microbiota in modulating multiple physiological barriers can be found in the review by Aburto et al. (2024) [27].

### 3.2. Regulation of Oxidative Stress and Neuroinflammation Through the Nrf2/HO-1 Pathway

As already stated, the brain is particularly vulnerable to oxidative stress [29]. In AD, oxidative stress is implicated in amyloid-β (Aβ) production and aggregation, as well as tau hyperphosphorylation and aggregation, thereby contributing to the development of the two major pathological hallmarks of the disease. In turn, Aβ and tau proteins further enhance ROS production, creating a self-sustaining vicious cycle that contributes to the progressive exacerbation of AD pathology [30].

**Figure 2:**
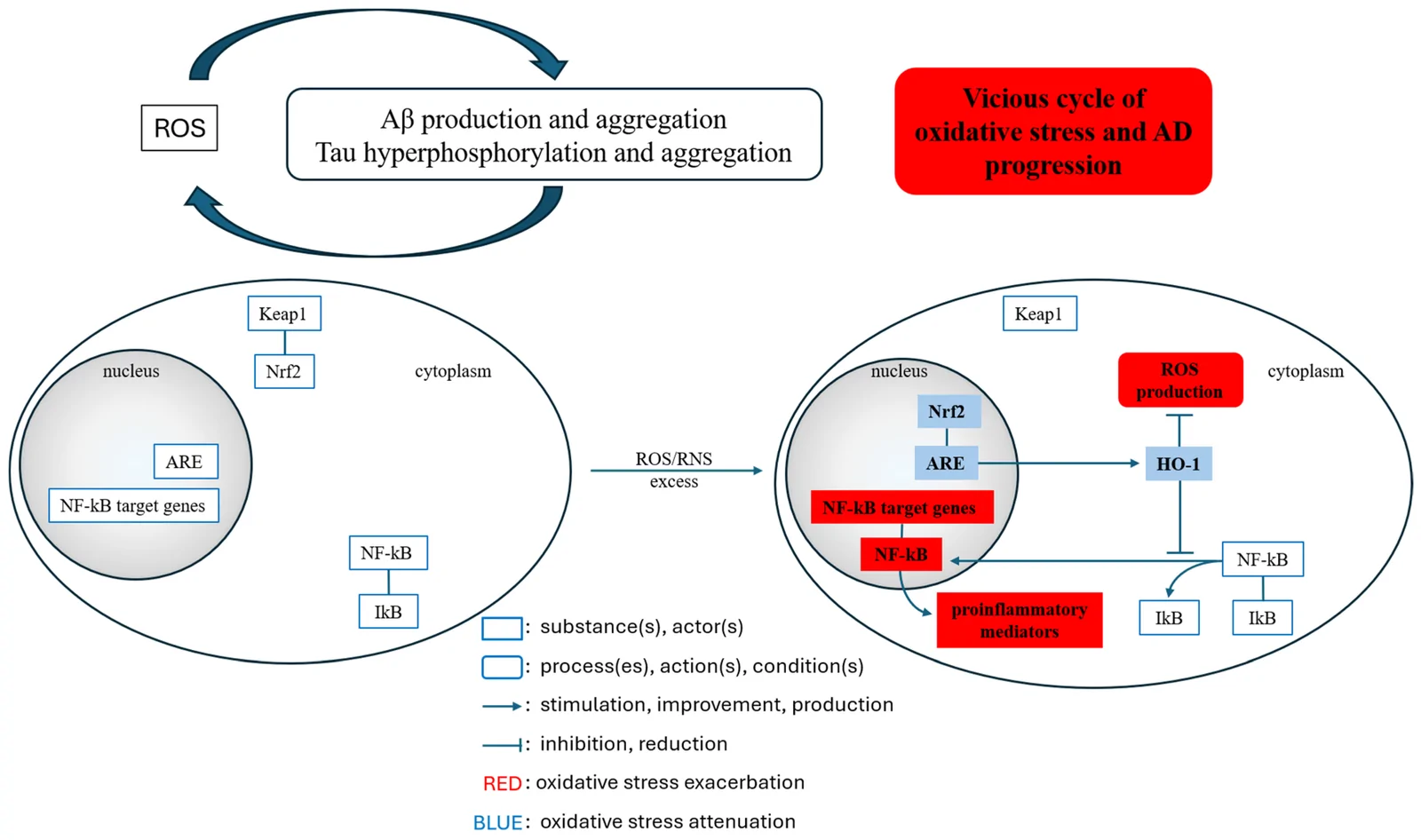
Schematic overview of Nrf2/HO-1-mediated regulation of oxidative stress and neuroinflammation: rectangles indicate substances or actors, whereas rounded rectangles indicate processes, actions, or conditions; solid arrows (→) indicate a positive regulatory effect, whereas T-bar arrows (⊣) indicate a negative regulatory effect; red and blue elements indicate oxidative stress exacerbation or attenuation, respectively. Abbreviations: AD, Alzheimer disease; ARE, antioxidant response element; Aβ, amyloid-β; HO-1, heme oxygenase-1; IκB, inhibitor of κB; Keap1, kelch-like ECH-associating protein 1; NF-κB, nuclear factor kappa-light-chain-enhancer of activated B cells; Nrf2, nuclear factor erythroid 2-related factor 2; ROS, reactive oxygen species; RNS, reactive nitrogen species. Authors’ original figure obtained through Microsoft PowerPoint.

Nuclear factor erythroid 2-related factor 2 (Nrf2) is a key transcription factor that, under basal conditions, is bound to its cytoplasmic inhibitor Kelch-like ECH-associating protein 1 (Keap1). In the presence of excessive ROS or RNS, Nrf2 dissociates from Keap1 and translocates into the nucleus, where it binds to the antioxidant response element (ARE), thus regulating the expression of antioxidant and anti-inflammatory genes [31]. In particular, the expression of heme oxygenase-1 (HO-1), a key downstream effector of Nrf2, represents an important mechanism in the anti-inflammatory crosstalk between Nrf2 and nuclear factor kappa-light-chain-enhancer of activated B cells (NF-κB) signaling pathways [32]. Furthermore, HO-1 activity contributes to the suppression of NF-κB-driven inflammatory responses, thereby reducing the expression of pro-inflammatory mediators [32]. The Nrf2/HO-1 axis in the dual regulation of oxidative stress and neuroinflammation is depicted in Figure 2.

### 3.3. Innate Immune Signaling and Inflammasome Activation

In NDs, chronic activation of innate immune signaling pathways is increasingly recognized as a major contributor to disease progression. Among these pathways, Toll-like receptor 4 (TLR4) plays a pivotal role in detecting both pathogen- and damage-associated molecular signals, thereby initiating inflammatory responses within the CNS. In the regulation of microbiota–gut–brain communication, TLR4 links peripheral dysbiosis to central neuroinflammation. Gut microbiota dysregulation leads to increased levels of pro-inflammatory microbial components, such as lipopolysaccharide (LPS), together with impaired intestinal barrier integrity, which facilitates the systemic translocation of these microbial-derived inflammatory mediators [33]. Once in the circulation, LPS interacts with TLR4 expressed on peripheral immune cells, leading to immune activation and amplification of pro-inflammatory cytokine production [34]. This systemic inflammatory state contributes to BBB dysfunction and altered brain immune signaling, thereby facilitating the transmission of peripheral inflammatory signals to the CNS [35].

Within the brain, microglia express TLR4 and are highly responsive to both LPS- and damage-related signaling pathways [36]. For example, in AD, proteolytic processing of amyloid precursor protein (APP) generates Aβ peptides, which aggregate and activate TLR4 signaling in microglia. Activation of microglial TLR4 initiates downstream myeloid differentiation primary response 88 (MyD88)-dependent signaling cascades, resulting in NF-κB activation [34] and the release of multiple pro-inflammatory mediators, including TNF-α, IL-6, cyclooxygenase-2 (COX-2), and inducible nitric oxide synthase (iNOS), which in turn enhances nitric oxide (NO) production and sustains the inflammatory environment. This sustained inflammatory response contributes to astrocyte activation, synaptic dysfunction, and neuronal damage [37]. The TLR4/MyD88 signaling axis functions as a central regulator of innate immune activation, neuroinflammation, and systemic immune–brain communication, and its persistent activation contributes to chronic neuroinflammatory states and is implicated in the progression of neurodegenerative processes [38]. This process corresponds to the priming step of the canonical two-step model of nucleotide-binding oligomerization domain (NOD)-like receptor family pyrin domain containing 3 (NLRP3) inflammasome activation, which is required to enable the inflammasome for subsequent activation. The second step, namely the activation phase, is triggered by cellular stress signals, such as ATP release, mitochondrial dysfunction, Aβ aggregates, and ROS production, leading to caspase-1 activation. Caspase-1 mediates the proteolytic maturation of pro-IL-1β into its active form, as well as pyroptotic cell death, a highly inflammatory form of programmed cell death characterized by membrane pore formation, cellular swelling, and release of intracellular pro-inflammatory contents [39,40]. This cascade further amplifies neuroinflammatory signaling, thereby sustaining a feed-forward loop of chronic inflammation.

**Figure 3:**
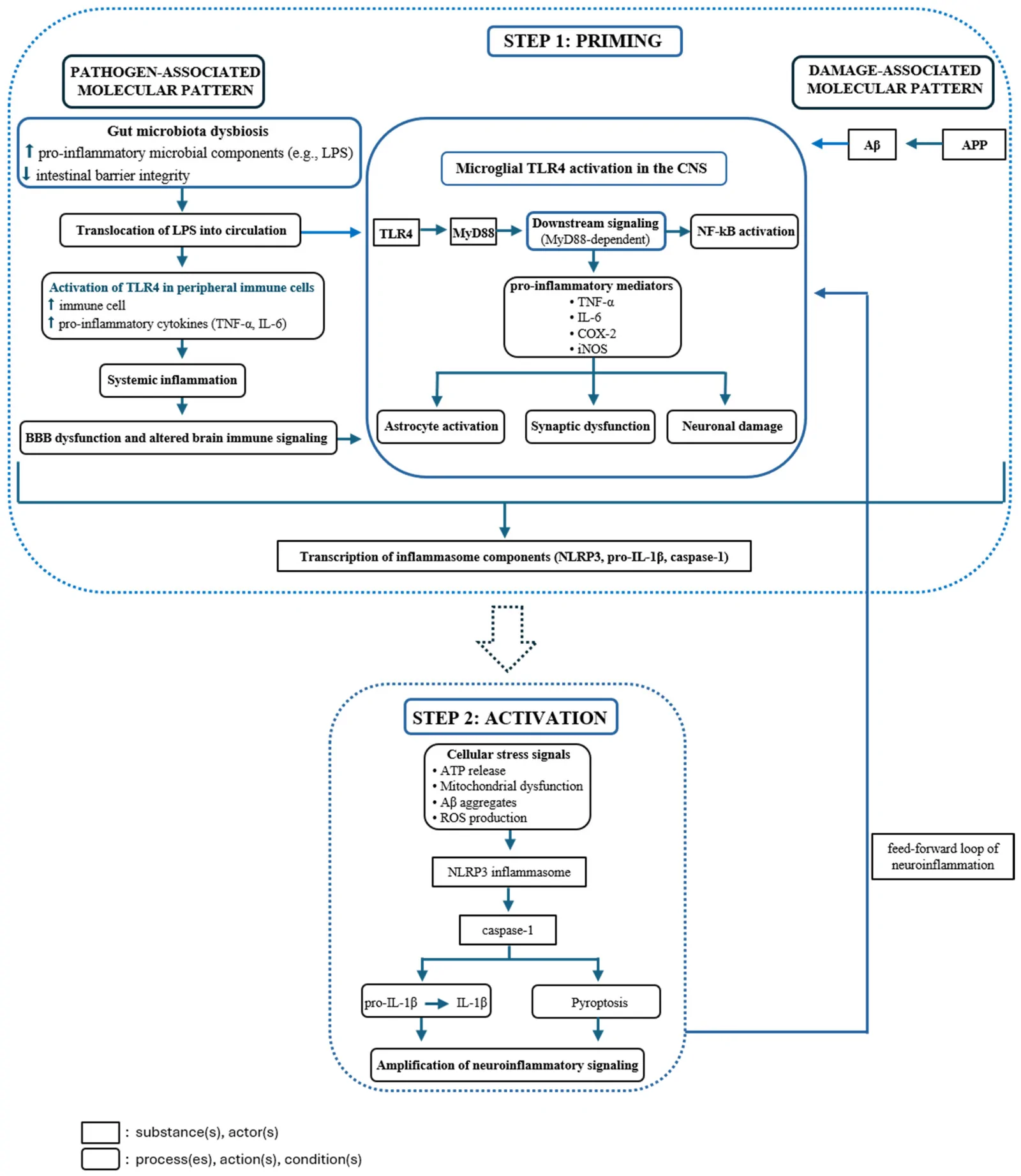
Schematic overview of TLR4-MyD88 signaling and NLRP3 inflammasome activation: rectangles indicate substances or actors; rounded rectangles indicate processes, actions, or conditions; upward arrows (↑) indicate an increase, whereas downward arrows (↓) indicate a decrease. Abbreviations: APP, amyloid precursor protein; ATP, adenosine triphosphate; Aβ, amyloid-β; BBB, blood–brain barrier; CNS, central nervous system; COX-2, cyclooxygenase-2; IL-6, interleukin-6; IL-1β, interleukin-1β; iNOS, inducible nitric oxide synthase; LPS, lipopolysaccharide; MyD88, myeloid differentiation primary response 88; NF-κB, nuclear factor kappa-light-chain-enhancer of activated B cells; NLRP3, NOD-like receptor pyrin domain containing 3; pro-IL-1β, pro-interleukin-1β; ROS, reactive oxygen species; TLR4, Toll-like receptor 4; TNF-α, tumor necrosis factor-α. Authors’ original figure obtained through Microsoft PowerPoint.

Overall, the mechanisms outlined above (see Figure 3) suggest that, within the MGBA, LPS not only activates TLR4 in peripheral immune cells but also enhances the expression of inflammasome components, thereby sensitizing both systemic and central immune compartments to secondary activation signals.

### 3.4. Modulation of Microglial M1/M2 Polarization

**Figure 4:**
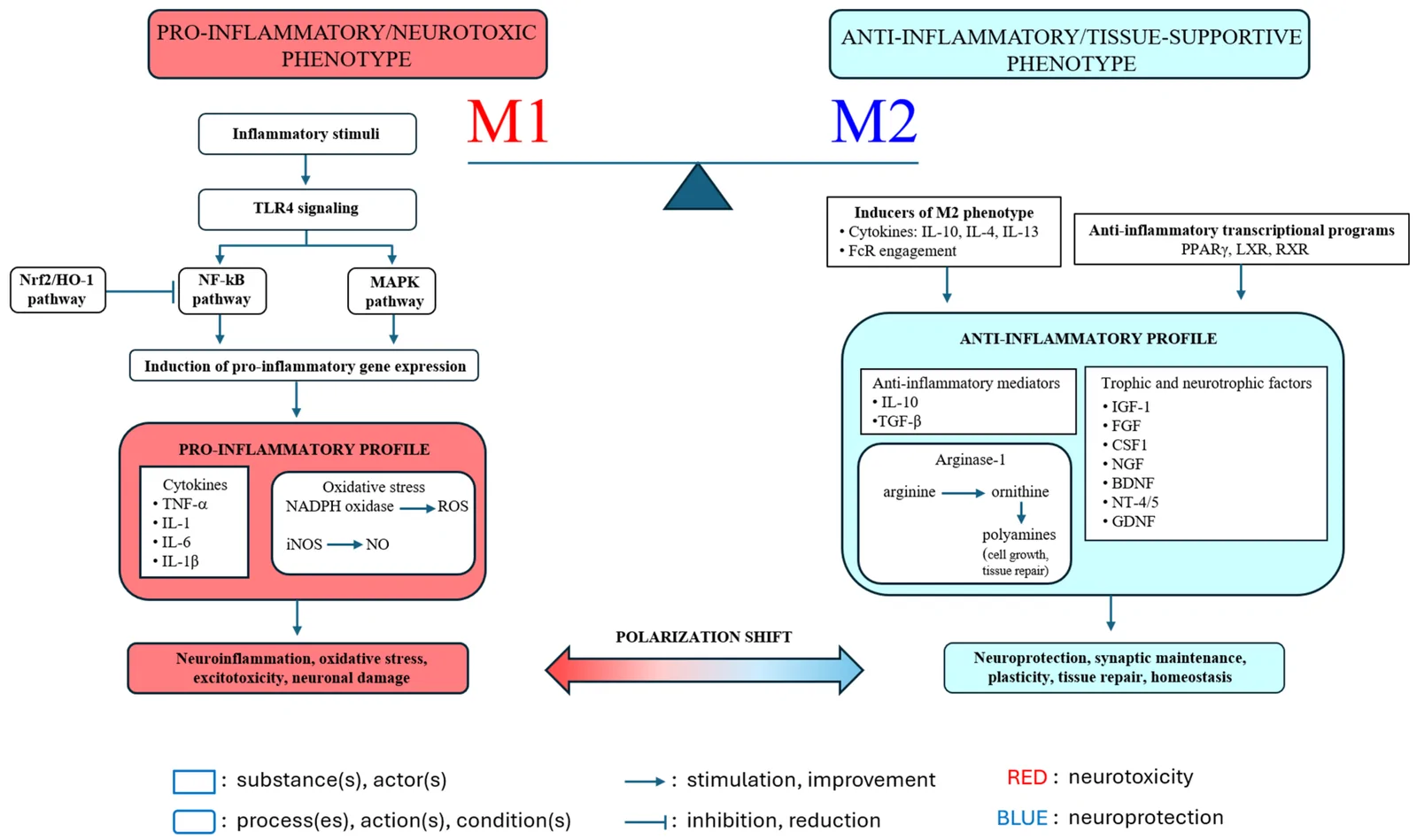
Schematic overview of modulation of microglial M1/M2 polarization: rectangles indicate substances or actors; rounded rectangles indicate processes, actions, or conditions; solid arrows (→) indicate a positive regulatory effect, whereas T-bar arrows (⊣) indicate a negative regulatory effect; red and blue elements indicate neurotoxicity or neuroprotection, respectively. Abbreviations: BDNF, brain-derived neurotrophic factor; CSF1, colony-stimulating factor 1; FcR, Fc receptor; FGF, fibroblast growth factor; GDNF, glial cell line-derived neurotrophic factor; HO-1, heme oxygenase-1; IGF-1, insulin-like growth factor 1; IL-1, interleukin-1; IL-4, interleukin-4; IL-6, interleukin-6; IL-10, interleukin-10; IL-13, interleukin-13; IL-1β, interleukin-1β; iNOS, inducible nitric oxide synthase; LXR, liver X receptor; MAPK, mitogen-activated protein kinase; NADPH, nicotinamide adenine dinucleotide phosphate; NF-κB, nuclear factor kappa-light-chain-enhancer of activated B cells; NGF, nerve growth factor; NO, nitric oxide; Nrf2, nuclear factor erythroid 2-related factor 2; NT-4/5, neurotrophin-4/5; PPARγ, peroxisome proliferator-activated receptor-γ; ROS, reactive oxygen species; RXR, retinoid X receptor; TGF-β, transforming growth factor-β; TLR4, Toll-like receptor 4; TNF-α, tumor necrosis factor-α. Authors’ original figure obtained through Microsoft PowerPoint.

The M1/M2 classification is a widely used but simplified heuristic model for describing pro- and anti-inflammatory features of microglial responses. However, it does not fully capture the heterogeneous, dynamic, and context-dependent spectrum of microglial activation states. Changes in the expression of M1- or M2-associated markers may indicate shifts in specific aspects of microglial responses but should not necessarily be interpreted as a complete transition from one phenotype to another. Nevertheless, microglial reprogramming from the pro-inflammatory M1 state toward the anti-inflammatory and tissue-supportive M2 phenotype may represent a critical and emerging mechanism of regulation in neuroinflammation and NDs (Figure 4) [41].

Inflammatory stimuli can activate Toll-like receptor 4 (TLR4)-mediated signaling, including downstream NF-κB [42] and mitogen-activated protein kinase (MAPK) pathways [43], thus promoting the expression of pro-inflammatory genes. Features commonly associated with the M1-like profile include increased production of pro-inflammatory cytokines and chemokines such as TNF-α, IL-1, IL-6, and IL-1β. Moreover, upregulation of nicotinamide adenine dinucleotide phosphate (NADPH) oxidase leads to increased production of ROS, while iNOS promotes NO generation, contributing to oxidative stress and excitotoxicity [44]. On the other hand, the Nrf2/HO-1 antioxidant pathway acts as a key counter-regulatory mechanism activated in response to oxidative stress and can attenuate inflammatory signaling, including NF-κB-associated responses, thereby contributing to redox homeostasis [45].

Conversely, features often associated with an M2-like profile include increased expression of anti-inflammatory mediators, including IL-10 and transforming growth factor-β (TGF-β), together with increased arginase-1 expression, which shifts arginine metabolism toward ornithine production rather than NO synthesis. Ornithine can serve as a precursor for polyamines involved in cellular growth and tissue repair. These responses may contribute to tissue-supportive functions, although their effects depend on the cellular and pathological context.

In parallel, M2-associated responses have also been linked to the production of trophic and neurotrophic factors such as insulin-like growth factor-1 (IGF-1), fibroblast growth factor (FGF), colony-stimulating factor-1 (CSF1), nerve growth factor (NGF), brain-derived neurotrophic factor (BDNF), neurotrophin-4/5 (NT-4/5), and glial cell line-derived neurotrophic factor (GDNF). These mediators can support neuronal survival, synaptic maintenance, and plasticity, in part through tropomyosin receptor kinase (Trk) receptor-mediated signaling [44]. The M2 phenotype can be induced by cytokines, such as IL-10, IL-4, and IL-13, as well as by Fc receptor (FcR) engagement (receptors that recognize the Fc portion of antibodies) [46], and can be regulated by transcriptional programs involving peroxisome proliferator-activated receptor-γ (PPARγ), liver X receptor (LXR), and retinoid X receptor (RXR) signaling pathways [44]. Notably, these markers and pathways reflect overlapping microglial responses rather than a distinct M2 state.

Summing up these findings, the polarization shift from an M1- to an M2-like phenotype may contribute to the promotion of tissue homeostasis and repair.

### 3.5. Mitochondrial Homeostasis and Neuroendocrine Regulation of the MGBA

Mitochondrial dysfunction also plays a central role in neuronal vulnerability and progression to NDs. In response to mitochondrial damage and impaired cellular energy metabolism, cells activate adaptive pathways aimed at restoring mitochondrial function, among which mitochondrial biogenesis represents a key compensatory mechanism.

**Figure 5:**
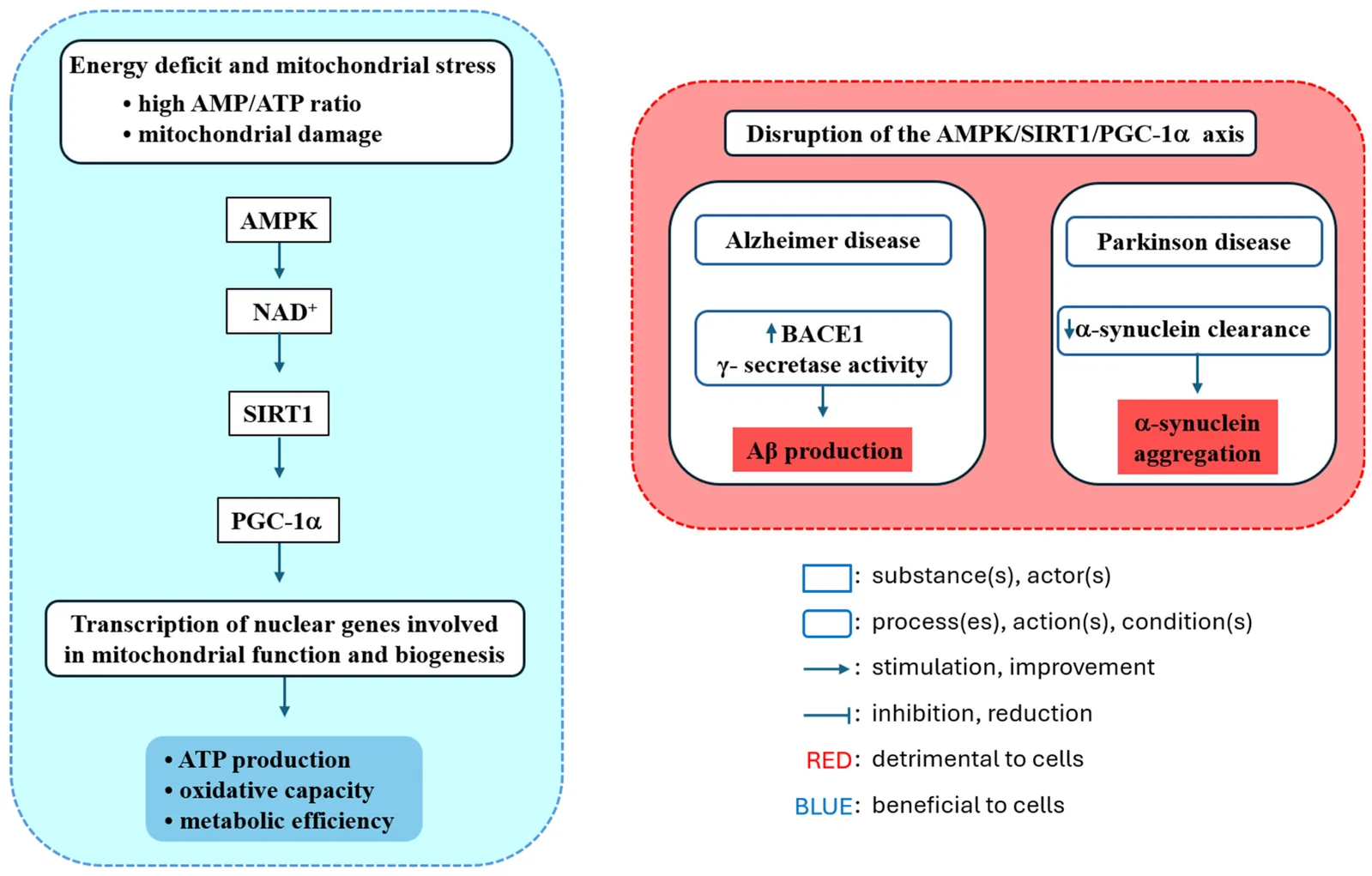
Schematic overview of AMPK/SIRT1/PGC-1α axis in neuronal health and disease: rectangles indicate substances or actors; rounded rectangles indicate processes, actions, or conditions; solid arrows (→) indicate a positive regulatory effect, whereas indicate a negative regulatory effect; red and blue elements indicate detrimental or beneficial effects to cells, respectively. Abbreviations: AMP, adenosine monophosphate; AMPK, AMP-activated protein kinase; ATP, adenosine triphosphate; Aβ, amyloid-β; BACE1, β-site APP-cleaving enzyme 1; NAD^+^, nicotinamide adenine dinucleotide; PGC-1α, PPAR-γ coactivator 1α; SIRT1, sirtuin 1. Authors’ original figure obtained through Microsoft PowerPoint.

The AMP-activated protein kinase (AMPK)/sirtuin 1 (SIRT1)/PPAR-γ coactivator 1α (PGC-1α) signaling axis (Figure 5) represents a central regulatory network linking cellular energy sensing to mitochondrial adaptation and metabolic efficiency [47]. When activated under conditions of cellular energy deficit, AMPK increases cellular NAD^+^ availability, thereby activating SIRT1, which in turn deacetylates and activates the transcriptional coactivator PGC-1α. This leads to the expression of nuclear genes involved in mitochondrial function and biogenesis. Disruption of this cascade may lead to Aβ production via β-site APP-cleaving enzyme 1 (BACE1)/γ-secretase in AD and impair α-synuclein clearance in PD [48]. Therefore, restoring normal AMPK/SIRT1/PGC-1α signaling could counteract mitochondrial dysfunction, a hallmark of neurodegeneration, and support neuronal survival and metabolic resilience.

**Figure 6:**
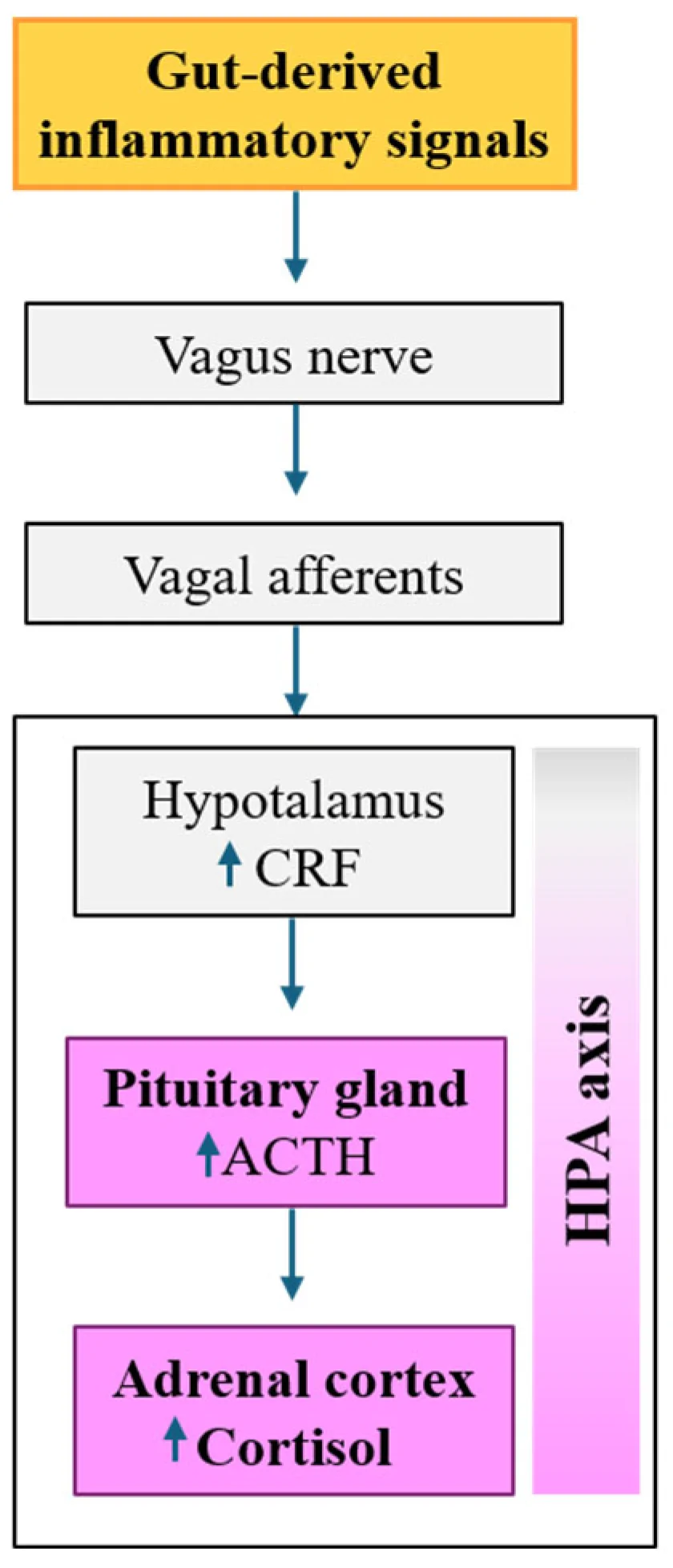
Schematic overview of vagus-nerve–HPA axis. Upward arrows (↑) indicate an increase in the substance level. Abbreviations: ACTH, adrenocorticotropic hormone; CRF, corticotropin-releasing factor; HPA, hypothalamic–pituitary–adrenal. Authors’ original figure obtained through Microsoft PowerPoint.

Emerging evidence further indicates that the relationship between the gut, the brain, and inflammation is mediated by the vagus nerve, whose afferent pathways also contribute to the regulation of the hypothalamic–pituitary–adrenal (HPA) axis (Figure 6). Under inflammatory conditions, the vagal afferent pathways activate the HPA axis, which stimulates the secretion of corticotropin-releasing factor (CRF) from the hypothalamus. CRF stimulates adrenocorticotropic hormone (ACTH) release from the pituitary gland, leading to the secretion of cortisol, the major stress hormone, from the adrenal cortex [49]. Therefore, activation of the HPA axis plays a key role in regulating neuroimmune and inflammatory responses, providing an additional pathway through which gut-derived signals can affect CNS function and neuroinflammatory processes.

### 3.6. Autophagy and Mitophagy as Regulators of Neuronal Proteostasis

In recent years, autophagy has also been found to be implicated in neurodegeneration. Autophagy is a lysosome-dependent degradative process responsible for the removal of damaged organelles, misfolded proteins, and aggregated proteins, thereby preventing their toxic accumulation within neurons. The cysteine protease cathepsin B (CatB) contributes to lysosomal degradation of autophagy substrates [50]. Conversely, neuronal autophagy enhances the turnover of BACE1, the principal neuronal β-secretase responsible for initiating APP cleavage and Aβ generation [51].

**Figure 7:**
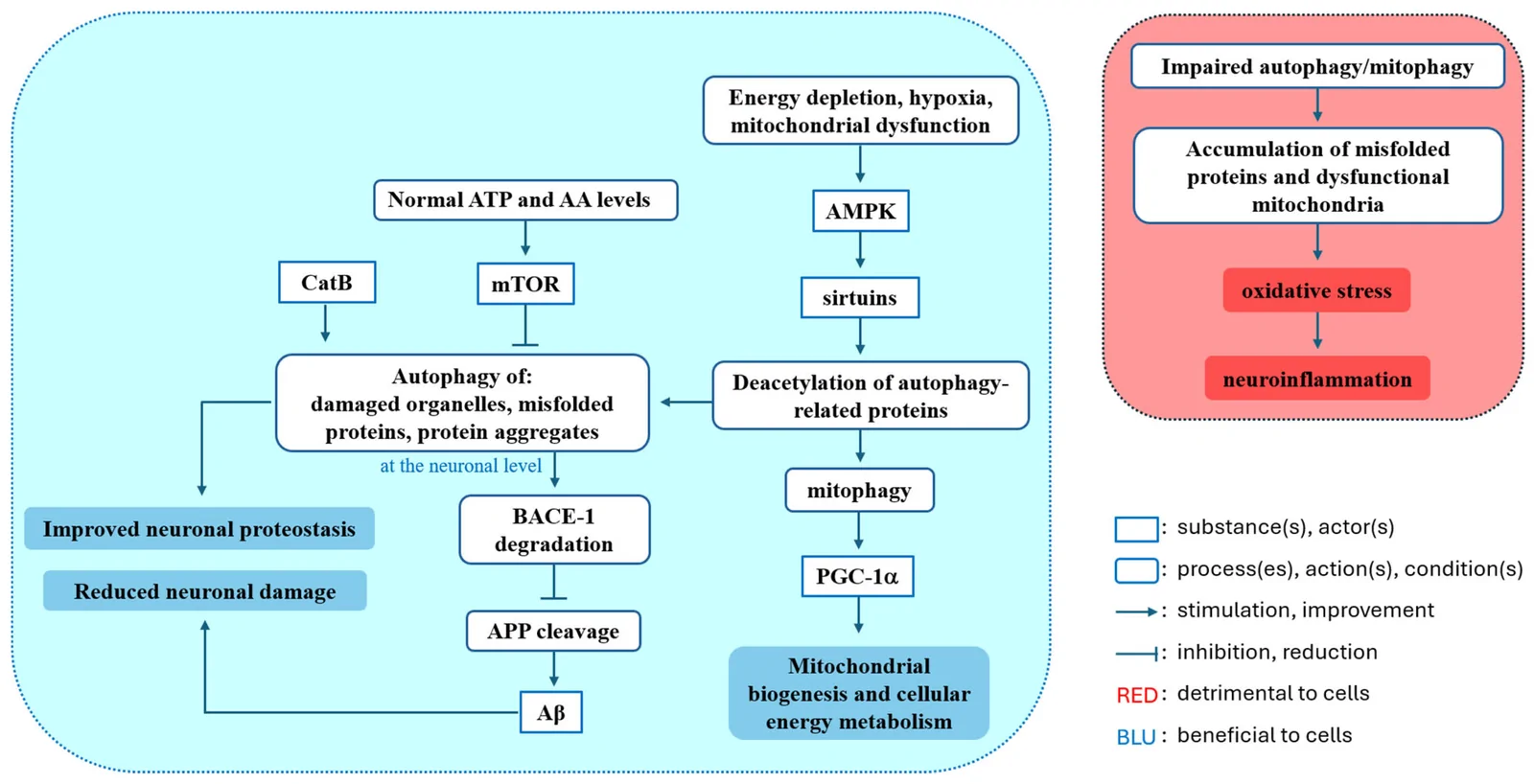
Schematic overview of regulation of neuronal proteostasis by autophagy and mitophagy: rectangles indicate substances or actors; rounded rectangles indicate processes, actions, or conditions; solid arrows (→) indicate a positive regulatory effect, whereas T-bar arrows (⊣) indicate a negative regulatory effect; red and blue elements indicate detrimental or beneficial effects to cells, respectively. Abbreviations: AA, amino acid; AMPK, AMP-activated protein kinase; APP, amyloid precursor protein; ATP, adenosine triphosphate; Aβ, amyloid-β; BACE1, β-site APP-cleaving enzyme 1; CatB, cathepsin B; mTOR, mechanistic target of rapamycin; PGC-1α, PPAR-γ coactivator 1α. Authors’ original figure obtained through Microsoft PowerPoint.

Specialized forms of autophagy also exist, the most studied being mitophagy, a selective autophagic process responsible for the removal of damaged or dysfunctional mitochondria [52,53]. Given that the brain is one of the most metabolically active organs in the body, deficits in mitochondrial quality control have been linked to numerous neurological and neurodegenerative disorders [54]. Accordingly, impaired mitophagy leads to the accumulation of dysfunctional mitochondria, contributing to neuronal dysfunction and the progression of NDs (Figure 7).

Autophagy is regulated by nutrient- and energy-sensing pathways involving the mechanistic target of rapamycin (mTOR), AMPK, and several members of the sirtuin family (SIRT1, SIRT3, SIRT6, and SIRT7). In the presence of normal levels of energy and amino acids, mTOR suppresses autophagic activity. Conversely, energy depletion, hypoxia, and mitochondrial dysfunction promote autophagy through AMPK activation and sirtuin-dependent deacetylation of autophagy-related proteins. These responses are accompanied by downstream activation of PGC-1α, a key regulator of mitochondrial biogenesis and cellular energy metabolism [52].

## 4. Natural Compounds as Modulators of MGBA in Alzheimer Disease

Natural compounds are an emerging class of bioactive modulators of the MGBA that act as signalling molecules capable of influencing gut microbial composition and host signalling pathways involved in neuroinflammation, BBB integrity, and the progression of NDs, particularly AD. Santhiravel et al. (2022) [55] provide a comprehensive review of the complex interplay between phytochemicals and the gut microbiota.

### 4.1. Direct Actions of Dietary Polyphenols in Modulating the MGBA

Among the phytochemicals investigated in the literature, polyphenols represent the most extensively studied class of compounds involved in MGBA modulation, and this topic has been comprehensively addressed in several recent reviews [56,57,58].

**Figure 8:**
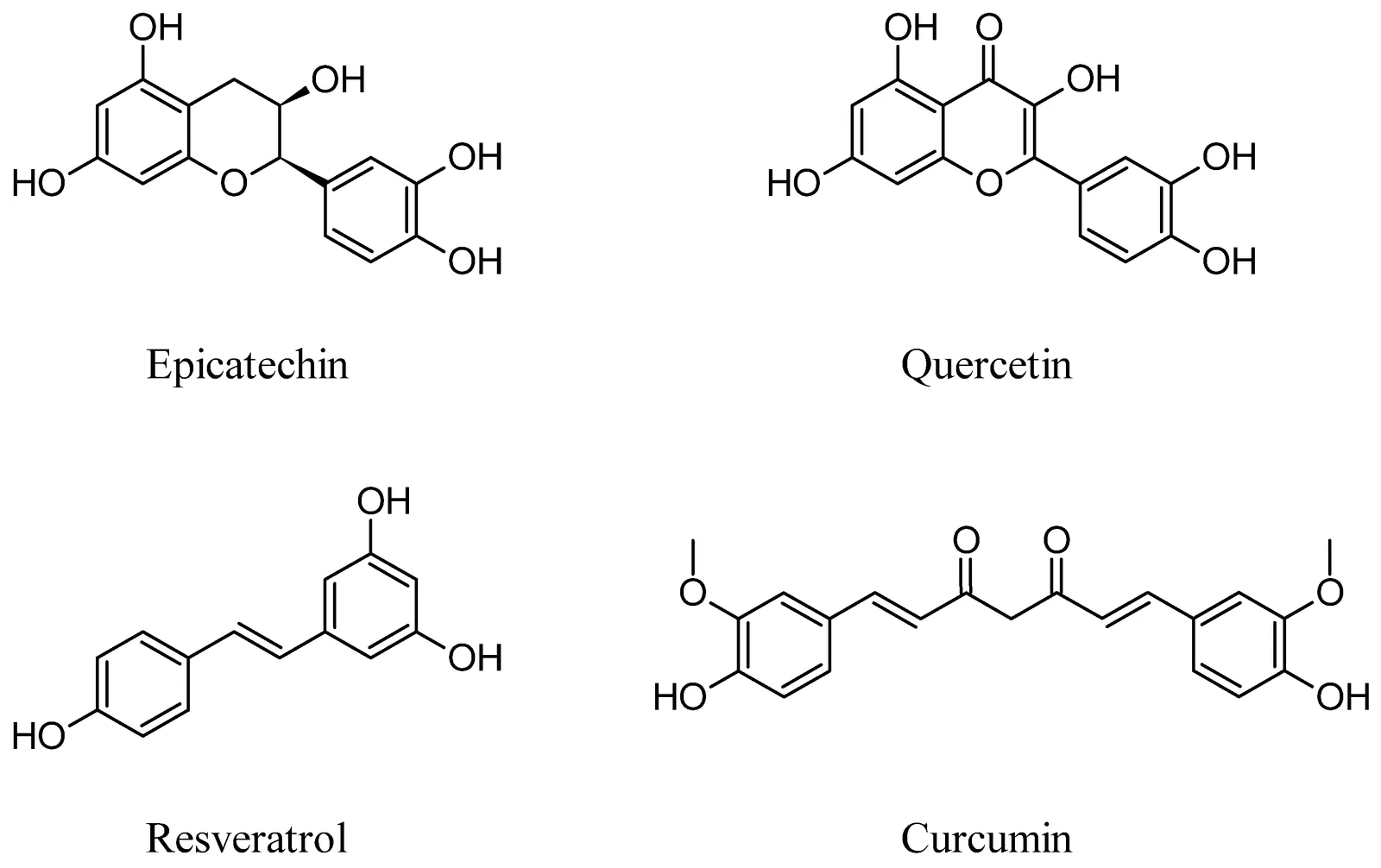
Chemical structures of representative natural polyphenols.

Polyphenols are naturally occurring compounds (see Figure 8) found in a wide range of dietary sources, including fruits, vegetables, cereals, tea, coffee, and wine. They exert neuroprotective effects through multiple pharmacological pathways and mechanisms. Owing to their limited intestinal absorption, a substantial fraction of dietary polyphenols bypasses absorption in the upper gastrointestinal tract and reaches the colon largely unchanged [59], where they interact with the gut microbiota and intestinal epithelium before exerting local and systemic biological effects.

Accumulating evidence indicates that polyphenols exert prebiotic-like effects, selectively promoting beneficial microbial taxa, including *Lactobacillus*, *Bifidobacterium* [60], *Akkermansia muciniphila*, and *Faecalibacterium prausnitzii* [61,62]. In parallel, they exert direct antimicrobial activity against pathogenic bacteria by disrupting bacterial cell membranes, interfering with bacterial energy metabolism, and chelating essential metal ions [63,64,65]. They also exert beneficial effects on tight junction integrity and gut epithelial barrier functions, as previously reviewed by Yang et al. (2016) [66].

In accordance with their well-established antioxidant activity [67], polyphenols exert protective effects against oxidative stress-induced BBB dysfunction. This protection is associated with reduced intracellular ROS levels, free radical scavenging activity, modulation of antioxidant enzyme expression, and chelation of metal ions involved in oxidative reactions, thereby contributing to the preservation of endothelial homeostasis and barrier integrity [68,69]. They also exert neuroprotective effects by modulating redox-sensitive signaling pathways, including Nrf2 and MAPK (among others), which regulate endogenous antioxidant responses and cellular protective gene expression [57].

Dietary polyphenols have been shown to influence microglial activation states through modulation of TLR4 signaling and its downstream effectors, including NF-κB and MAPK pathways. This regulation is associated with attenuation of pro-inflammatory microglial responses and a shift toward an M2-like phenotype. Accordingly, polyphenols reduce the expression and release of inflammatory mediators such as TNF-α, IL-1β, and IL-6 while also promoting the expression of anti-inflammatory and neuroprotective factors [70]. This polarization shift is further supported by the activation of redox-sensitive pathways, particularly the Nrf2/HO-1 signaling pathway, triggered by dietary polyphenols, thus contributing to reduced oxidative stress and reinforcement of the anti-inflammatory and neuroprotective microglial phenotype [71]. In this connection, the role of polyphenols as modulators of microglial polarization in NDs has been recently reviewed [41,72,73,74].

Polyphenols can also modulate autophagy and mitophagy by regulating multiple signaling pathways, including AMPK, SIRT1, and mTOR, thereby contributing to the maintenance of cellular and mitochondrial homeostasis [75,76]. For more detailed information on the role of natural products targeting mitochondrial dysfunction in neurological disorders, the reader is referred to the review by Liang et al. (2021) [77].

### 4.2. Gut Microbiota-Derived Bioactive Metabolites from Dietary Components and Their Role in the MGBA

Metabolites produced by the gut microbiota from dietary components constitute a broad class of bioactive compounds that include, but are not limited to, short-chain fatty acids (SCFAs), a wide range of polyphenol-derived microbial metabolites, and amino acid-derived metabolites.

#### 4.2.1. Short-Chain Fatty Acids as Key Microbial Metabolites of Dietary Fiber

**Figure 9:**
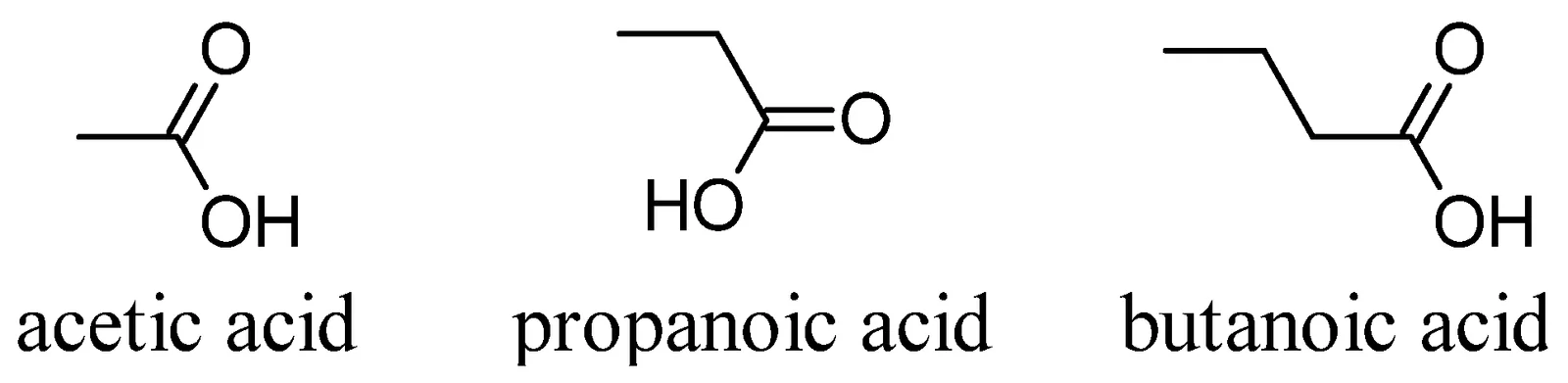
Chemical structures of representative SCFAs.

SCFAs, primarily acetate, propionate, and butyrate (Figure 9), are the major end-products of gut microbial saccharolytic fermentation of non-digestible carbohydrates (e.g., dietary fibers and oligosaccharides derived from whole grains, legumes, fruits, and other plant-based foods) [78]. They inhibit the growth of enteric pathogens in the intestine [79] and, at low concentrations (1 mM), also enhance intestinal epithelial barrier function by stimulating mucin 2 (MUC2) expression, a key marker of mucus-secreting cell function and mucosal barrier integrity [80].

SCFAs play a pivotal role in regulating immune homeostasis and inflammatory responses. Among the major products, butyrate increases the expression of tight junction proteins, including claudin-1 and ZO-1, and promotes occludin redistribution [81], thus improving intestinal barrier integrity.

SCFAs also modulate microglial function, thereby contributing to the regulation of neuroinflammatory responses. They exert direct anti-inflammatory effects on microglia, attenuating the inflammatory response in primary microglial cultures by inhibiting HDAC activity and NF-κB activation, and suppressing LPS-induced inflammatory signaling. In particular, 100 μM acetate, butyrate, or their combination, under LPS-stimulated conditions, suppresses both HDAC and NF-κB activity, leading to reduced gene expression of *TNF* (encoding TNF-α) and, consequently, decreased secretion of TNF-α, along with a broader downregulation of pro-inflammatory cytokines such as IL-1β and IL-6. Reduced inflammatory response of microglia was also observed in vivo in mice fed with dietary fiber, which increased SCFA levels in the cecum and in the hepatic portal circulation. These findings suggest that SCFAs produced by the gut microbiota may reach the circulation and contribute to the regulation of microglial inflammatory responses [82].

A comprehensive overview of the role of SCFAs in gut–brain communication is provided by Silva et al. (2020), which also discusses G protein-coupled receptor (GPCR) signaling, including GPR41 and GPR43 (renamed FFAR3 and FFAR2, respectively) [83].

Beyond restoring microbial balance, accompanied by improved intestinal barrier function and reduced systemic inflammation, and modulation of microglial maturation, SCFAs also preserve BBB function via GPCR activation, HDAC inhibition, and modulation of NF-κB and Nrf2 signaling pathways. These effects have been observed across different in vitro and in vivo experimental models and under diverse experimental conditions, not limited to neurodegenerative diseases [84]. These findings suggest that SCFAs exert coordinated effects across the MGBA, spanning from the intestinal lumen to the regulation of CNS homeostasis.

Finally, microbial-derived butyrate is the primary energy source for colonocytes, preventing them from undergoing autophagy [85].

#### 4.2.2. Polyphenol-Derived Gut Microbial Metabolites

Beyond fiber-derived fermentation products, the gut microbiota metabolizes dietary polyphenols into a diverse pool of low-molecular-weight metabolites that act as key bioactive mediators of neuroprotection in NDs. The biological activity and production of these metabolites are strongly influenced by interindividual differences in gut microbial composition and enterotype-specific metabolic capacity [86]. Among the most extensively characterized polyphenol-derived microbial metabolites are phenolic acids, phenyl-γ-valerolactones (PVLs), and urolithins. In particular, phenolic acids arise from the microbial degradation of a broad range of dietary polyphenols, PVLs are generated from flavan-3-ols, and urolithins derive from ellagitannins and ellagic acid. Growing evidence suggests that these metabolites contribute substantially to the biological effects traditionally attributed to their parent compounds, often with improved bioavailability, greater systemic exposure, and distinct biological activities.

##### *Phenolic* *Acids*

**Figure 10:**
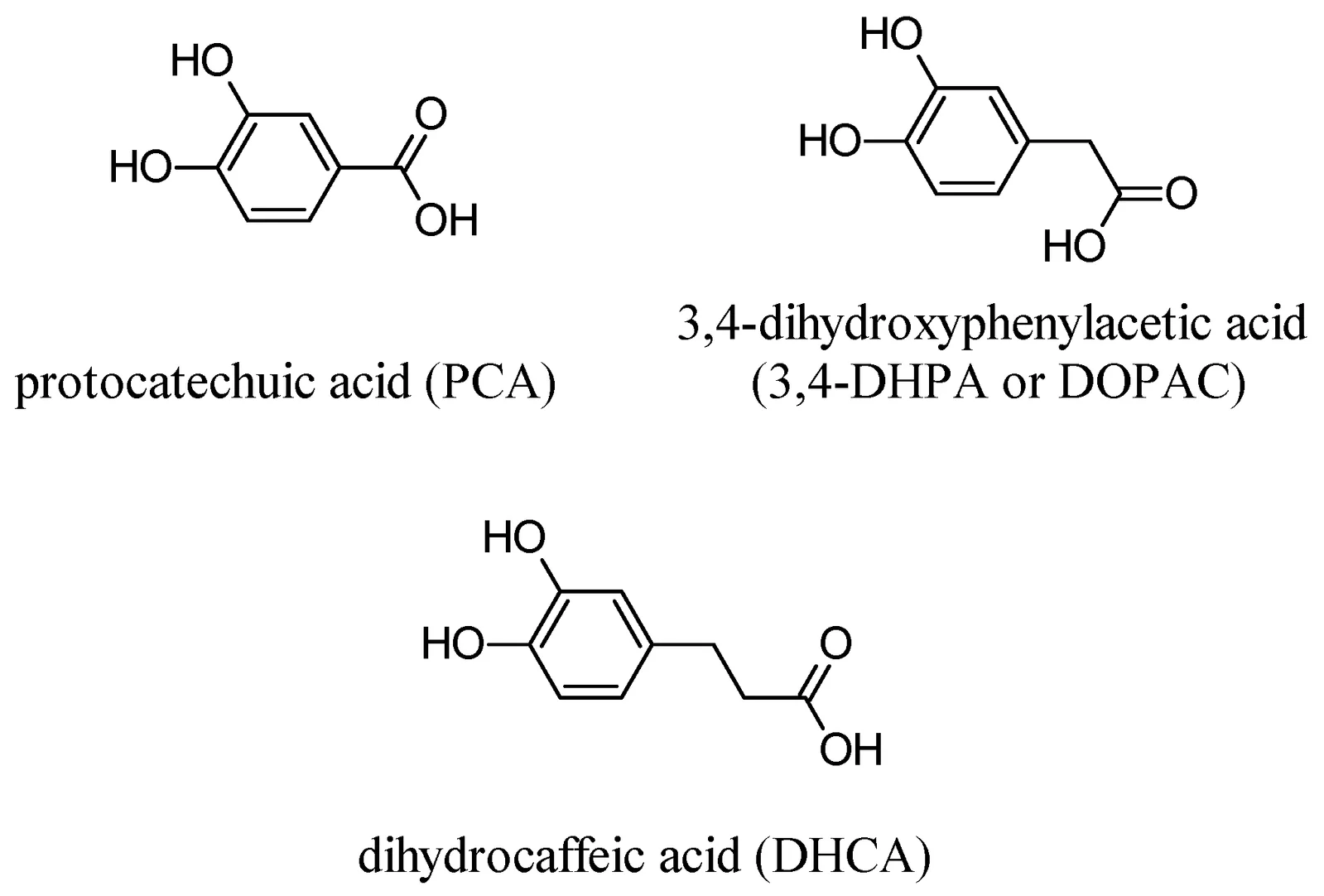
Chemical structures of the most representative microbial-derived phenolic acids.

Among the most extensively studied microbial-derived phenolic acids (or phenolcarboxylic acids) (Figure 10) are protocatechuic acid (PCA), 3,4-dihydroxyphenylacetic acid (3,4-DHPA), and dihydrocaffeic acid (DHCA). PCA deserves particular attention, as it is not only a major microbial metabolite generated from the biotransformation of several dietary polyphenols but also naturally present in several plant-derived foods, including berries, red wine, fruits, and vegetables.

It has been demonstrated that PCA exerts antibacterial activity through ROS-mediated lipid peroxidation and DNA fragmentation in bacterial cells [87]. In general, phenolic acids maintain intestinal barrier integrity through the upregulation of tight junction protein expression, modulation of mucus secretion, inhibition of oxidative stress and inflammatory responses, and regulation of gut microbiota composition [88].

Furthermore, PCA may attenuate NLRP3 (NOD-, LRR- and pyrin domain-containing protein 3) inflammasome activation predominantly through upstream modulation of oxidative stress and NF-κB signaling. It has been proposed to modulate redox homeostasis through its potent antioxidant and free radical-scavenging properties [89], as well as by enhancing endogenous antioxidant defenses via upregulation of key antioxidant enzymes, such as superoxide dismutase (SOD) and catalase (CAT). These effects have been observed in rat pheochromocytoma (PC12) cells [90], a tumor-derived cell line commonly used to investigate neuronal responses to neurotoxic stimuli. However, findings obtained in this model should be interpreted with caution, as PC12 cells do not fully reproduce the phenotype and functional complexity of primary neurons.

Furthermore, PCA has been shown to inhibit ROS overproduction and prevent glutathione depletion in intestinal Caco-2 cells [91]. Furthermore, PCA activates the Nrf2 signaling pathway in J774A.1 macrophages, promoting the upregulation of glutathione peroxidase (GPx) and glutathione reductase (GR) [92]. These mechanisms enhance cellular antioxidant defenses, improve glutathione recycling, restore redox homeostasis, and protect cells against oxidative damage.

In both LPS-stimulated RAW 264.7 macrophages and BV2 microglial cells, phenolic acids exert anti-inflammatory effects by modulating key pro-inflammatory mediators, particularly TNF-α, IL-6, and IL-8. In LPS-stimulated RAW 264.7 macrophages, for example, free PCA, 3,4-DHPA, and DHCA, along with their conjugated derivatives, have been shown to reduce the secretion of these cytokines, an effect associated with decreased oxidative stress as evidenced by reduced intracellular ROS levels [93]. In agreement with these findings, PCA attenuates inflammatory responses in LPS-stimulated BV2 microglia by modulating TLR4-dependent NF-κB and MAPK signaling pathways, thereby reducing the production of the aforementioned cytokines as well as prostaglandin E_2_ (PGE_2_) [94].

Beyond its effects on oxidative stress and inflammation, PCA has also been reported to interfere with pathological protein aggregation by inhibiting the aggregation of Aβ and α-synuclein (α-Syn) and promoting the destabilization of their preformed fibrils. Furthermore, PCA protects PC12 cells from Aβ- and α-synuclein-induced cytotoxicity, thereby preventing neuronal cell death associated with these neurotoxic protein species [95].

Finally, PCA promotes the autophagy–lysosomal pathway, alleviating neuronal damage in in vivo and in vitro models of AD [96], and promotes mitophagy, leading to improvement in mitochondrial dysfunction [97].

Overall, the ability of phenolic acids, and PCA in particular, to modulate redox homeostasis, attenuate neuroinflammatory signaling, and interfere with the aggregation of neurotoxic proteins underscores their potential as multi-target neuroprotective agents for the prevention and management of NDs.

##### *Phenyl-γ-Valerolactones* (PVLs)

**Figure 11:**
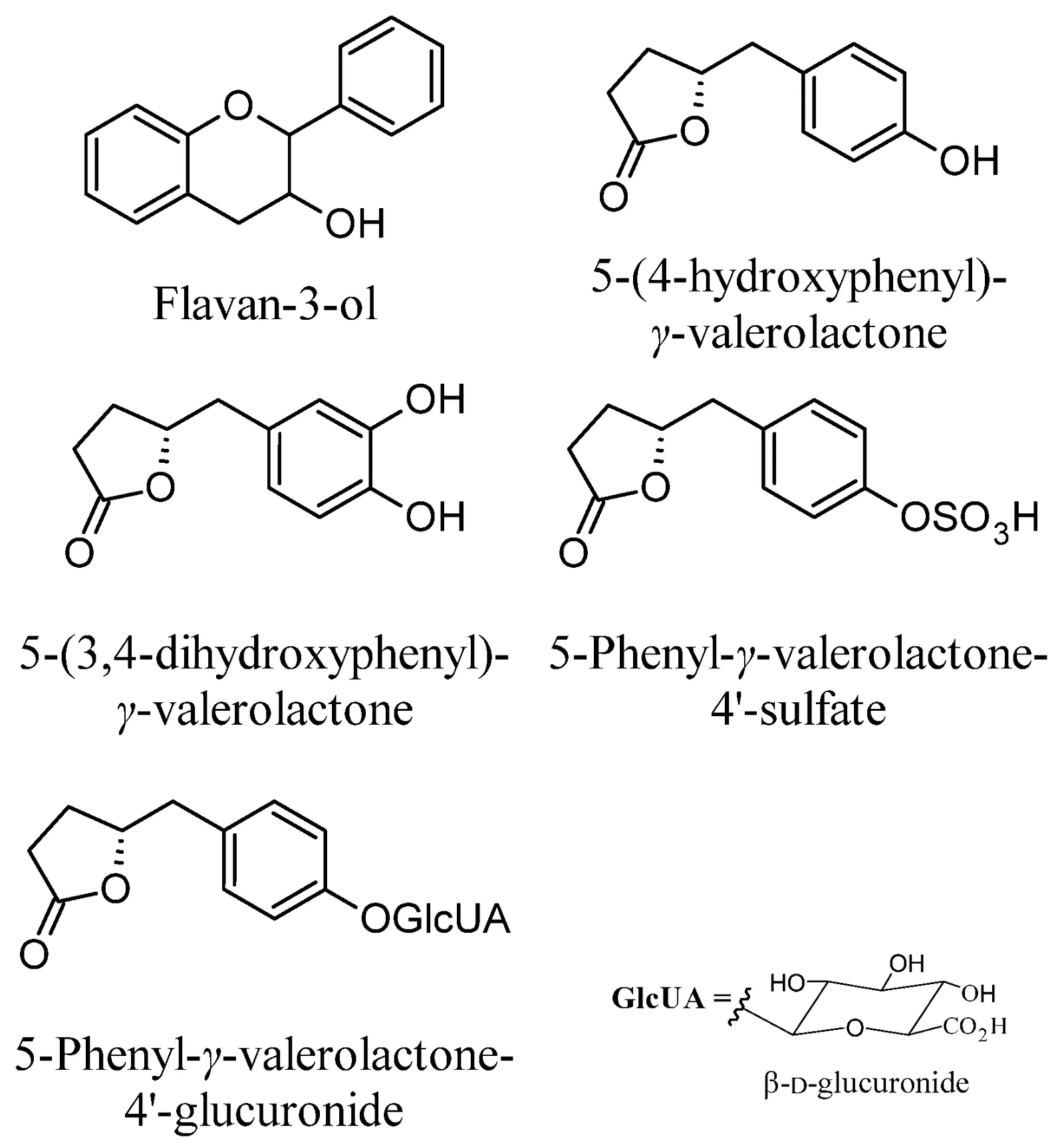
Chemical structures of phenyl-γ-valerolactones as microbial metabolites of flavan-3-ol.

PVLs (Figure 11) are gut microbiota-derived metabolites generated from flavan-3-ols, a subclass of flavonoids abundant in cocoa, tea, and other plant-derived foods.

Unlike their parent flavonoids, PVLs appear to be capable of crossing the BBB and reaching the CNS, as recently demonstrated for 5-(4-hydroxyphenyl)-γ-valerolactone sulfate in both in vitro and in vivo models [98]. Beyond their demonstrated CNS bioavailability, increasing attention has been devoted to their pharmacological activities within the brain. Although the literature investigating their role in AD and other NDs remains relatively limited, a growing body of evidence has begun to elucidate their neuroprotective potential through both in vitro and in vivo studies.

Recent evidence supports the anti-inflammatory potential of 5-(3,4-dihydroxyphenyl)-γ-valerolactone, alone and in combination with curcumin, in primary rat cortical microglia stimulated with LPS, as demonstrated by a reduction in the production of key pro-inflammatory mediators, including IL-1β, TNF-α, and NO. The anti-inflammatory activity was attributed to the downregulation of NLRP3 expression. Moreover, in microglia, the combination of 5-(3,4-dihydroxyphenyl)-γ-valerolactone and curcumin significantly reduced the mRNA expression of NADPH oxidase (NOX2)-dependent ROS production while enhancing the expression of two key Nrf2 target genes, such as HO-1 and NAD(P)H quinone dehydrogenase 1 (NQO-1), which are responsible for the expression of antioxidant and neuroprotective genes [99].

PVLs appear to modulate Aβ pathology through complementary mechanisms acting at different stages of protein metabolism and aggregation. In particular, they counteract APP-induced impairments of the proteasome–autophagy system in neuronal SH-SY5Y cells, a tumor-derived model widely used to investigate neuronal processes but with limitations in reproducing the properties of primary neurons. They modulate cellular proteolysis through proteasome inhibition and compensatory autophagy upregulation, while also inhibiting cathepsin B activity, thereby reducing misfolded protein accumulation and proteostasis-related stress [100]. Consistent with these findings, in vivo studies further support the neuroprotective role of PVLs. These compounds counteract Aβ oligomer-induced synaptic impairment and memory deficits in a mouse model of AD [101], suggesting a beneficial effect on cognitive performance and synaptic plasticity.

Recent multi-omic analyses have also shown that 5-(4-hydroxyphenyl)-γ-valerolactone modulates the response of TNFα-stimulated human brain microvascular endothelial cells. In particular, its sulfate and glucuronide conjugates alter gene expression programs involved in cell adhesion, cytoskeletal organization, and barrier permeability, thus promoting a less inflammatory and less permeable endothelial phenotype [102].

Overall, PVLs influence several processes implicated in neurodegeneration, including Aβ pathology, neuroinflammatory signaling, endothelial function, and proteostasis. Hence, they have the potential to exert cognitive and neuroprotective effects when consumed as flavan-3-ol-rich foods.

##### *Urolithins* 

**Figure 12:**
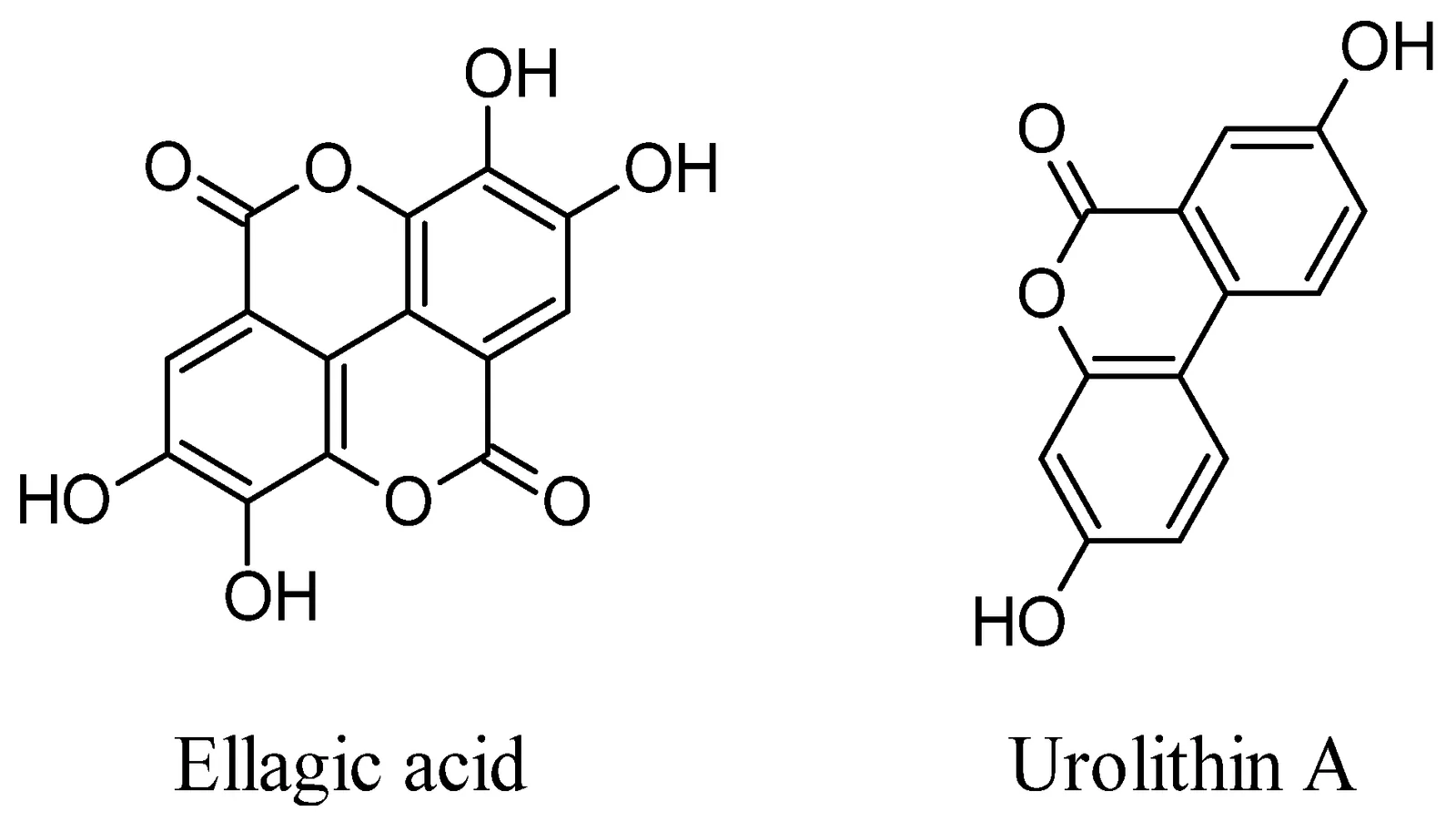
Chemical structure of ellagic acid and urolithin A.

Ellagitannin- and ellagic acid-rich foods such as pomegranate, berries, and walnuts yield urolithins through gut microbial metabolism [103,104], with urolithin A (Figure 12) being a metabolite of growing interest in health-related research.

Urolithin A modulates intestinal barrier function by enhancing mucus secretion through the activation of Nrf2 and AhR pathways and favors SCFA-producing bacteria [105,106].

In general, urolithins exhibit potent antioxidant activity by reducing ROS production [107], thus limiting oxidative damage to neuronal macromolecules such as lipids, proteins, and DNA. In particular, urolithin A reduces excessive colonic inflammation in preclinical models of colitis [106].

Moreover, urolithin A exerts anti-inflammatory effects in both RAW264.7 cells and primary murine peritoneal macrophages, reducing the production of key pro-inflammatory mediators, including TNF-α and IL-6, while simultaneously suppressing iNOS expression. In these macrophage models, urolithin A attenuates NF-κB signaling by preventing IκB degradation, subsequent NF-κB nuclear translocation, and its DNA binding. In addition, it inhibits AP-1 (activator protein-1) activation by suppressing c-Jun nuclear translocation and modulates both phosphoinositide 3-kinase/protein kinase B (PI3K/Akt) and c-Jun N-terminal kinase (JNK) signaling pathways. The resulting reduction in NADPH oxidase (NOX)-dependent ROS production contributes to the attenuation of inflammatory signaling and cytokine release [108]. Although these findings provide mechanistic insights into the anti-inflammatory effects of urolithin A in macrophages, they cannot be directly extrapolated to microglial responses.

Urolithin A also inhibits NLRP3 inflammasome activation in macrophages stimulated with the canonical NLRP3 activator, nigericin. In particular, it suppresses inflammasome assembly and downstream effector responses, including caspase-1 activation and IL-1β maturation and secretion, while reducing both intracellular and mitochondrial ROS accumulation. The ROS-dependent interaction between thioredoxin-interacting protein (TXNIP) and NLRP3 is therefore inhibited, thus further preventing inflammasome activation. These findings have been corroborated in an NLRP3-driven mouse model of peritonitis, in which urolithin A attenuates inflammasome-mediated inflammation [109].

At the mitochondrial level, urolithin A promotes cellular homeostasis primarily by inducing mitophagy through the activation of energy-sensing signaling pathways, including AMPK and the SIRT1/PGC-1α axis. Specifically, SIRT1 regulates mitofusin-2 (Mfn2) expression, a mitochondrial outer membrane GTPase involved in mitochondrial homeostasis, via PGC-1α-dependent transcriptional programs. AMPK activation and concomitant mTOR inhibition further promote mitophagy induction, thus contributing to the maintenance of mitochondrial homeostasis [110].

Beyond its effects on inflammation and mitochondrial function, urolithin A also improves cognitive performance, reduces neuronal apoptosis, and enhances neurogenesis in the APP/PS1 mouse model of AD by enhancing cerebral AMPK activation and decreasing the activation of key inflammatory pathways, including NF-κB and MAPK signaling. Furthermore, it modulates amyloid pathology through downregulation of BACE1 expression, resulting in reduced cerebral Aβ production and deposition. In parallel, plaque-associated inflammatory responses, including microgliosis and astrocytosis in both the cortex and hippocampus, are attenuated. Together, these effects are associated with improved neuronal integrity and cognitive function in APP/PS1 mice [111]. Finally, some urolithins also inhibit Aβ fibrillation and aggregation, as demonstrated by the thioflavin T (ThT) binding assay [112].

Overall, by modulating oxidative stress, inflammation, mitochondrial homeostasis, mitophagy, and amyloid pathology, urolithin A emerges as a promising multi-target bioactive compound for mitigating AD pathogenesis and promoting neuronal survival.

#### 4.2.3. Tryptophan-Derived Indole Metabolites

**Figure 13:**
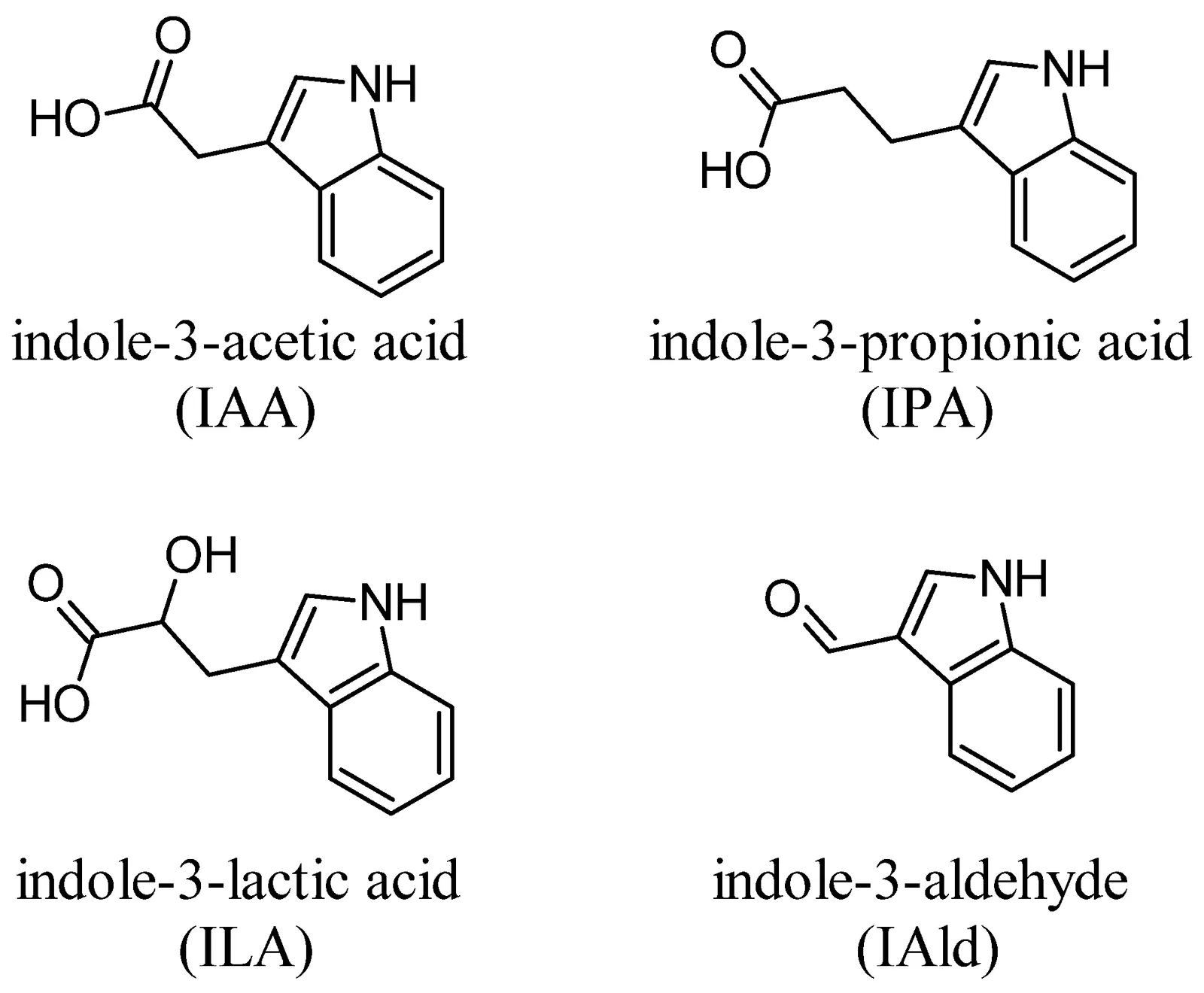
Representative structures of indole derivatives.

Indole derivatives arise from microbial metabolism of dietary tryptophan, an amino acid abundant in soy products, legumes, seeds, and fermented foods. Among these metabolites, indole-3-acetic acid (IAA or I3A), indole-3-propionic acid (IPA), indole-3-lactic acid (ILA), and indole-3-aldehyde (IAld) (Figure 13) are the most extensively studied in the context of host–microbiota interactions and MGBA signaling.

These metabolites can regulate epithelial barrier integrity and immune homeostasis at the intestinal mucosal interface. First, a mixture of indole, IAA, and IPA has been shown to reverse gut barrier injury in APP/PS1 mice by increasing the expression of the tight junction proteins occludin and ZO-1 [113]. Furthermore, IAld and ILA modulate mucosal immune homeostasis via AhR-dependent pathways. IAld promotes AhR-dependent IL-22 transcription, and the resulting IL-22-mediated mucosal response observed in mice supports epithelial homeostasis and promotes the coexistence of diverse microbial communities in the intestinal mucosa [114]. Similarly, ILA activates AhR in intestinal CD4^+^ T cells, promoting their differentiation into immunoregulatory CD4^+^CD8αα^+^ T cells by downregulating the transcription factor ThPOK, a key regulator of CD4 T-cell identity. Reduced ThPOK expression enables partial reprogramming toward a CD8αα^+^ intraepithelial-like phenotype endowed with regulatory functions, thereby contributing to intestinal immune homeostasis [115]. In parallel, IPA acts as a PXR ligand, strengthening intestinal barrier function by reducing enterocyte TNF-α expression and promoting tight junction gene expression, in coordination with TLR-dependent immune signaling pathways [116]. This barrier-stabilizing activity limits the translocation of microbial products into the systemic circulation, thereby reducing peripheral inflammatory burden.

Beyond their local effects on the intestinal mucosa, microbiota-derived indole metabolites also modulate inflammatory responses in peripheral immune and metabolic tissues, including macrophages and hepatocytes. These findings suggest that indole signaling contributes to systemic immune regulation, linking gut microbial metabolism with peripheral inflammation and brain health. In particular, IAA and tryptamine reduce pro-inflammatory cytokine production induced by fatty acids and LPS and inhibit macrophage migration. Moreover, IAA attenuates lipid-induced inflammatory responses in hepatocytes and downregulates genes involved in lipid biosynthesis. Notably, these effects are largely mediated through AhR signaling [117]. IPA also exhibits potent antioxidant and neuroprotective activities, including hydroxyl radical scavenging and protection against oxidative tissue damage. Experimental studies have also shown that IPA protects neuroblastoma cells from Aβ-induced toxicity and oxidative stress triggered by SOD inhibition or hydrogen peroxide exposure [118]. Moreover, IPA has been shown to exert neuroprotective effects in experimental models of cerebral ischemia by reducing ROS-mediated injury, lipid peroxidation, and oxidative DNA damage. It also suppresses ischemia-induced astrocyte and microglial activation, thereby attenuating neuroinflammatory responses [119].

Emerging evidence further indicates that microbiota-derived indole metabolites can act directly within the CNS, thus completing the functional loop of the MGBA. In fact, IPA and IAld, among other microbiota-derived tryptophan metabolites, attenuate inflammatory responses in astrocytes by activating the type I interferon (IFN-I)/AhR signaling axis, reducing the expression of pro-inflammatory genes such as *Ccl2* and *Nos2*, and promoting a more homeostatic astrocyte phenotype [120]. IAA and IPA improve cognitive performance in APP/PS1 mice by reducing Aβ aggregation and tau hyperphosphorylation. These effects are accompanied by increased expression of synaptic plasticity-related proteins, including postsynaptic density protein 95 (PSD-95) and synapsin I, together with attenuation of excessive microglial reactivity, as evidenced by a reduced number of ionized calcium-binding adaptor molecule 1 (Iba-1)-positive cells (with Iba-1 being a specific marker protein for microglial reactivity). Furthermore, they upregulate AhR expression, reduce NF-κB activation and COX-2 expression, and finally inhibit NLRP3 inflammasome assembly [113]. Furthermore, IPA has been shown to improve cognitive function and reduce Aβ production and accumulation through neuronal PXR activation, leading to suppression of NF-κB signaling and downregulation of BACE1 [121]. In a randomized, double-blind, placebo-controlled multicenter trial involving healthy older adults, probiotic supplementation was associated with increased circulating IPA levels and a favorable neurotrophic and inflammatory profile. In complementary in vitro experiments, IPA reduced TNF-α production in activated microglia, while neuronal cells exposed to conditioned media from IPA-treated microglia showed increased production of BDNF and NGF, which are involved in neuronal survival and synaptic plasticity. These experimental findings support the potential anti-inflammatory and neurotrophic properties of IPA, although their relevance to CNS target engagement and therapeutic efficacy in AD remains to be established [122]. Finally, in a model of sepsis-associated encephalopathy, IPA restored BNIP3L-dependent mitophagy in hippocampal neurons by inhibiting BNIP3L ubiquitination, thus improving mitochondrial homeostasis and reducing neuronal injury [123].

For a more comprehensive discussion of the emerging neuroprotective properties of IPA, readers are referred to the recent review by Owe-Larsson et al. (2025) [124]. An overview of natural compounds as modulators of the MGBA in NDs is presented in Table 1.

## 5. Medicinal Chemistry Optimization of Microbial Metabolites

Microbial metabolites are considered privileged scaffolds for drug discovery because they are produced in a biological context in which they participate in interactions with microbial communities and, in some cases, the host. As products of evolution through natural selection, they possess structural features relevant to molecular recognition, although these do not necessarily imply superior safety or tolerability. Indeed, some microbial metabolites display physicochemical characteristics often associated with drug-like compounds, such as a relatively high number of sp^3^-hybridized carbon atoms (Csp^3^), a limited aromatic proportion, and chirality centers [127]. Nevertheless, their drug-like behavior in humans at therapeutically relevant doses remains to be established.

Despite growing evidence supporting the systemic effects of microbial metabolites, their potential clinical use in the treatment of NDs is limited by pharmacokinetic and pharmacodynamic drawbacks. At first, several metabolites are chemically unstable because they contain readily hydrolyzable or oxidable functional groups, thus conceivably undergoing extensive peripheral metabolism and rapid systemic clearance before reaching the CNS. Reduced oral bioavailability is therefore expected. Moreover, most polar compounds display poor BBB permeability. Therefore, a medicinal chemistry optimization process could lead to metabolite derivatives with improved drug-like properties, taking inspiration from the natural product tributyrin (tributyryl glycerol), a naturally occurring prodrug of butyric acid endowed with more favorable pharmacokinetic properties compared with butyrate itself [128]. In 2024, Williams and Cao reported on SCFAs and tryptophan prodrugs, among which the butyrate prodrug butyroyloxymethyl diethylphosphate (AN-7) showed cardioprotective activity associated with HDAC inhibition and increased HO-1 expression. This result raises the possibility of investigating this compound in neurodegenerative disorders, where HDAC-dependent epigenetic regulation and the Nrf2/HO-1 antioxidant pathway are implicated in neuroprotection [129]. Moreover, another butyrate prodrug, namely pivaloyloxymethyl butyrate (AN-9), was described as capable of improving survival and growth in a mouse model of spinal muscular atrophy. However, whether this effect results from sufficient concentrations of butyrate becoming available in the CNS after oral AN-9 administration or is mediated by its activation of a gut–CNS axis mechanism remains to be established [129]. Finally, tyrosol-SCFA esters delay SCFA release until they reach the distal GI tract and improve oral SCFA bioavailabilities by virtue of their membrane-crossing abilities [129]. Pharmaceutical technology approaches, including enteric encapsulation and nanoformulations, have been explored to overcome the pharmacokinetic limitations of SCFAs and tryptophan-derived microbial metabolites, but the examples discussed by Williams and Cao do not specifically concern the treatment of neurodegenerative diseases [129].

Pharmacodynamic limitations also constrain the clinical translation of microbial metabolites. The microbial metabolites generally display relatively low binding affinities—they are often reported in the literature as weak ligands. Thus, they would require the administration of high systemic doses to achieve therapeutic efficacy. Since their targets are widely expressed throughout the body, systemic administration of high doses could increase the risk of off-target and adverse effects. On the other hand, most microbial metabolites fulfill the Rule of Three and might be intended as fragments [130]. Microbial biomimicry has therefore emerged in recent years as a promising medicinal chemistry strategy aimed at developing potent drug-like molecules possibly endowed with reduced ADMET liabilities.

The first investigation was conducted by Saha et al. (2016) [131], who searched for the occurrences of metabolite mimics in the compound collection of GlaxoSmithKline (GSK). They identified 421 ‘metabolite mimics’, including SCFAs as the largest class, flavonoids and nonflavonoid (urolithin A) derivatives, and indole derivatives, some of which had previously been tested in target-based assays and displayed activity against 101 different human targets, including enzymes and GPCRs.

**Figure 14:**
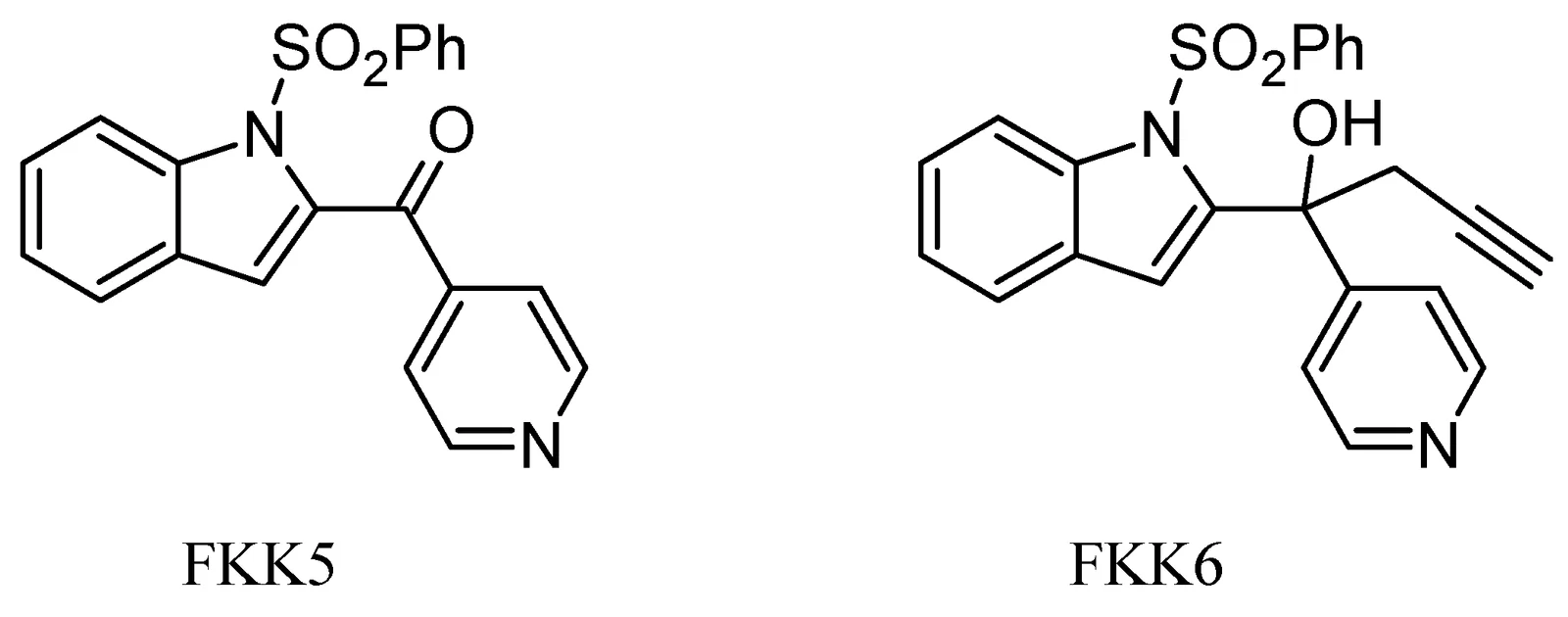
Structures of FKK5 and FKK6.

Further evidence supporting microbial metabolite mimicry as a drug discovery strategy was provided by Dvořák and colleagues, who identified two indole-derived hit compounds, namely FKK5 and FKK6 (Figure 14), as nontoxic PXR ligands. They shed light on the mimicry of AhR and PXR ligand metabolites derived from microbes as lead compounds endowed with anti-inflammatory properties [132]. An in-depth survey of microbial metabolite mimics in drug discovery, covering the limited literature available in this emerging field, was published by the same authors in 2020 [133].

Recently, urolithin A has also been exploited as a scaffold for medicinal chemistry optimization, leading to a series of 8-hydroxy-modified derivatives investigated as phosphodiesterase 2 (PDE2) inhibitors. The most active compound was computationally predicted to have a favorable BBB permeability profile compared with the well-known PDE2 inhibitor BAY60-7550 [134]. However, we could not find experimental proof of its actual BBB permeability behavior.

Furthermore, IPA was used as a scaffold for the design of arylhydrazone hybrids with multifunctional neuroprotective properties. The in vitro BBB model showed that some compounds increased the permeability of the endothelial monolayer while maintaining the tight junctions [135].

**Figure 15:**
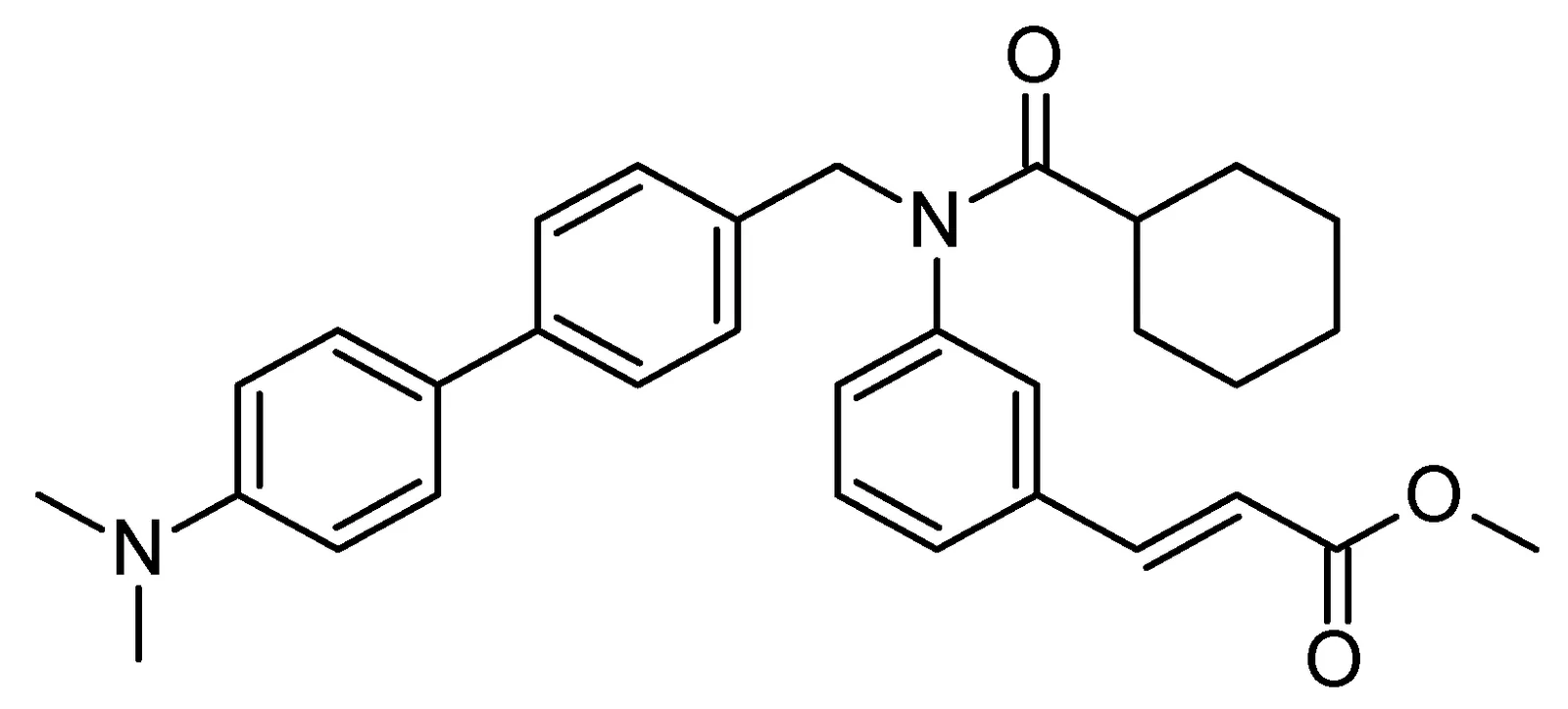
Structure of fexaramine.

It is noteworthy that the ability of microbial metabolites to modulate multiple molecular targets involved in coordinated biological responses is increasingly considered a therapeutic advantage (polypharmacology, multipotency), rather than a limitation, when these interactions do not result in off-target effects or unpredictable biological outcomes. It has been suggested that highly selective and potent drugs could be responsible for a high risk of triggering compensatory or redundant biological pathways or of disrupting physiological homeostasis, as illustrated by tyrosine kinase inhibitors and selective COX-2 inhibitors [136]. In contrast, an ideal drug should be inspired by our microbiota, which modulates multiple metabolic pathways to restore and sustain the homeostasis of the biological system. This idea has inspired so-called ‘metabolic bionics-based drug discovery’ as a new medicinal chemistry optimization approach aimed at allowing a more effective balance of biological effects within the organism rather than reaching increased potency or target selectivity. Zheng and coworkers report the PXR ligand FKK6 for gut inflammatory disorders and SCFA analogs for adipocyte differentiation as examples of this approach. They also suggest the development of gut-restricted drugs for remote regulation, with the advantage of minimizing off-target effects. The intestinally restricted FXR agonist fexaramine (Figure 15) and intestinally targeted TGR5 agonists are reported as examples of this approach. Notably, the antidepressant effects obtained by reducing the production of segmented filamentous bacteria-derived autoinducer-2, leading to a reduction in intestinal Th17 cell generation (a subset of T helper cells producing pro-inflammatory cytokines) and their hippocampal accumulation, demonstrate that targeting intestinal pathways can produce CNS effects, thus supporting the development of microbial metabolite analogs as a promising strategy for the treatment of NDs [136].

## 6. General Summary and Conclusions

NDs are multifactorial disorders characterized by several common pathological mechanisms including, among others, oxidative stress, mitochondrial dysfunction, neuroinflammation, and impaired proteostasis, which are further influenced by the MGBA. Unfortunately, currently available therapies remain predominantly symptomatic and do not modify disease progression.

Emerging evidence indicates that natural bioactive compounds, particularly dietary polyphenols, and their microbial metabolites exert pleiotropic effects across multiple levels of the MGBA, thus displaying potential neuroprotective relevance in NDs. Notably, rather than directly or indirectly influencing the brain, these compounds modulate gut microbial composition and functionality, transforming the microbiota into a dynamic metabolic organ capable of producing metabolites with systemic and neuroactive properties [137]. Through this integrated network, they influence intestinal barrier integrity, oxidative stress and neuroinflammation, innate immune signaling and inflammasome activation, microglial polarization, mitochondrial homeostasis, and neuronal proteostasis, collectively contributing to the maintenance of neurobiological homeostasis. The available evidence encompasses different levels of experimental validation, from cell-free in vitro assays to cell-based in vitro studies and in vivo models. For example, PCA has been shown to attenuate NLRP3 inflammasome and oxidative stress signaling in cellular models [90,91,92,93,94]. Similarly, 5-(3,4-dihydroxyphenyl)-γ-valerolactone showed antioxidant and anti-inflammatory effects in LPS-stimulated primary rat cortical microglia [99], urolithin A reduced NLRP3 inflammasome activation in LPS-primed BMDMs [109], IPA has been shown to exert neuroprotective effects in experimental models of cerebral ischemia [118,119], and IAld attenuates pro-inflammatory responses in astrocyte models [120]. In vivo evidence of neuroprotection has also been reported. PCA has been reported to alleviate neuronal damage by promoting the autophagy–lysosomal pathway [97], urolithin A improves cognitive performance and modulates amyloid pathology and neuroinflammation in APP/PS1 mice [111], and IPA has also shown neuroprotective effects in different in vivo models [113,120,121]. Notably, the biological effects observed following direct administration of individual compounds should not be automatically interpreted as microbiota-mediated, as the endogenous microbial production, physiological exposure, and microbiota dependence of these effects may not have been established. Accordingly, the extent to which the proposed mechanisms are directly demonstrated, are indirectly supported, or remain hypothetical should be considered when interpreting the available evidence.

Overall, the current evidence is predominantly preclinical, comprising mechanistic hypotheses, biochemical and cellular findings, and efficacy data from animal models. Human studies provide preliminary associations but do not establish a causal relationship between microbiota-derived metabolites and clinical outcomes in AD. Moreover, the available evidence is insufficient to establish the long-term safety of these compounds for therapeutic use. Finally, no disease-modifying benefit in humans has been observed from the evidence reviewed. Further pharmacokinetic, translational, and clinical studies are required to establish CNS exposure, target engagement, therapeutic efficacy, optimal bioavailability, and long-term safety in human populations.

## Figures and Tables

**Table 1:** Natural compounds and microbiota-derived metabolites as modulators of MGBA in AD.

**Compounds**	**Origin/Precurs or**	**Gut Microbiota Modulation ^a^**	**Preservation of Barrier Integrity ^a,b^**	**Oxidative Stress Regulation ^a^**	**Anti-Inflammatory Pathways ^a^**	**Mitochondrial Homeostasis/Autophagy ^a^**	**Systemic and CNS Exposure ^a^**
Dietary polyphenols (e.g., epicatechin, quercetin, resveratrol, curcumin) ^c^	Fruits, vegetables, cereals, tea, coffee, wine	prebiotic-like effects; [60,61,62] ^d,e^; antibacterial activity [63,64,65] ^d^	↑tight junction proteins [66] ^d^	direct and indirect antioxidant activity [68,69] ^d^; Nrf2 and MAPK modulation [57] ^d^	↓ TLR4-dependent NF-κB and MAPK signaling pathways [70] ^d^; M2 polarization [72,74] ^d^ (quercetin: 5 μM, in vitro, human microglial cells HMC3; 60 mg/kg/day, 49 days, in vivo, male C57BL/6 mice with Aβ_1–42_-induced AD) [73]	modulation of multiple pathways [76] ^d^	low bioavailability, instability in the gastrointestinal tract, and fast metabolism [57]; poorly absorbed in the small intestine and do not achieve physiologically relevant concentrations in circulation [112]
Short-chain fatty acids (SCFAs) (acetate, propionate, butyrate) ^f^	Microbial saccharolytic fermentation of non-digestible carbohydrates	antibacterial activity [79] ^d^	↑MUC2 expression (butyrate: 1 mM, in vitro, LS174T colonic cancer cell line) [80] ^e^; ↑ tight junction proteins (butyrate: 4 mM, in vitro, cdx2-IEC intestinal cells) [81] ↑ GPCR, ↑ Nrf2, ↓ HDAC, ↓ NF-κB [84] ^d^	indirect reduction of oxidative stress	↓ HDAC and NF-κB (acetate/butyrate: 100 μM, in vitro—LPS-stimulated primary mouse microglia; ex vivo, 2.5% inulin diet, 8 weeks) [82]	energy source for colonocytes (butyrate: 10 mM, ex vivo, colonocytes isolated from germ-free mice) [85]	systemic exposure: SCFAs are metabolized in the colonocytes; a minor fraction reaches the systemic circulation and other tissues [83,84]. CNS exposure: oral administration of SCFAs drives maturation of microglia in germ-free mice (indirect evaluation) [83]; after injection of ^14^C-butyrate into the carotid artery of rats, the concentration of butyrate in the brain reached 46% of the plasma level [84]; butyrate detected in the human brain [84]
Phenolic acids (PCA, 3,4-DHPA/DOPAC, DHCA) ^f^	Microbial degradation of dietary polyphenols ^g^	restored gut microbial diversity [88] ^d^; antibacterial activity (PCA: 1.2–2.2 μg/mL, in vitro, bacterial cultures of *E. coli*, *P. aeruginosa*, and *S. aureus* –ATCC strains) [87]	↑tight junction proteins, ↓ inflammatory factors, ↓ oxidative stress [88] ^d^	direct and indirect antioxidant activity [88] ^d^ (phenolic acids: 0.05–0.10 mg/mL, in vitro, cell-free chemical assay) [89]; (PCA: 1.2 mM, in vitro, PC12 cells—MPP^+^-induced neurotoxicity) [90]; (PCA: 0.25 mM, in vitro, Caco-2 cells) [91]; (PCA: 25 μM, in vitro, macrophage cell line J774 A.1) [92]	modulation of pro-inflammatory mediators (phenolic metabolites: 1–50 μM, in vitro, RAW 264.7 murine macrophages) [93]; ↓ TLR4-dependent NF-κB and MAPK signaling pathways (PCA: 5–20 μM, in vitro, BV2 murine microglial cells) [94]	mitophagy and autophagy activation (PCA: 50–400 μM, in vitro, primary hippocampal neurons; in vivo, 50 mg/kg/day blueberry extract, 16 weeks, APP/PS1 transgenic mice) [96] (PCA: 40 μM, in vitro, neural stem cells; 10–60 mg/kg/day, 7 days, in vivo, MCAO/R model) [97]	Systemic exposure: phenolic acids reach the systemic circulation mainly as conjugated metabolites, with concentrations varying depending on the compound, the food matrix, and the gut microbiota [88]; studies in both animal models and humans demonstrated that PCA is absorbed and reaches systemic circulation as a major metabolite of anthocyanins; PCA can be detected in various organs, including brain tissue [96]. CNS exposure: the effects on the brain are likely mediated by indirect mechanisms [88,96,97]
Phenyl-γ-valerolactones (PVLs) ^f^	Microbial metabolites of flavan-3-ols (abundant in cocoa, tea, and other plant-derived foods)	– ^h^	– ^i^ modulation of pathways involved in endothelial permeability and brain endothelial cell integrity (sulphate/glucuronide of 4-hydroxy PVL: 0.65/0.35 μM, in vitro, TNF-α-stimulated human brain microvascular endothelial cells) [102]	↓ NOX2-dependent ROS; ↑ HO-1 and NQO-1 (3,4-dihydroxy PVL: 1–50 μM, in vitro, LPS-stimulated primary rat cortical microglia) [99]	↓ NLRP3 expression; modulation of NOX2/Nrf2 signaling pathway (3,4-dihydroxy PVL: 1–50 μM, in vitro, LPS-stimulated primary rat cortical microglia) [99]	proteasome-autophagy regulation; cathepsin B activity inhibition (PVLs: 0–10 μM, in vitro, SH-SY5Y cells) [100]	Systemic exposure: PVLs reach the bloodstream 4–24 h after flavan-3-ols intake, accounting for 42% of ingested (–)-epicatechin [98]; 5-(3,4-dihydroxyphenyl)-γ-valerolactone has been identified in plasma and urine, with concentrations in the low micromolar range [99] CNS exposure: in silico analyses based on QPlogBB suggest that many PVLs may be able to cross the BBB; 5-(3,4-dihydroxyphenyl)-γ-valerolactone sulfate was identified in the brain tissue of rats and pigs by spectrometric analysis [98]
Urolithins ^f^	Microbial metabolites of ellagitannins/ellagic acid (abundant in pomegranate, berries, and walnuts)	↑ SCFA-producing bacteria (urolithin A: 20–100 mg/kg twice daily, 7 days, in vivo, male C57BL/6 mice) [105]	Nrf2/AhR-mediated barrier protection (urolithin A: 10 μM, in vitro, LS174T cells; 100 mg/kg twice daily, 7 days, in vivo, male C57BL/6 mice) [105]; (urolithin A: 50 μM, in vitro, HT29 and Caco-2 human colon epithelial cells; 50 μM, ex vivo, colon explants from C57BL/6; 20 mg/kg/day, 7 days, in vivo, C57BL/6 mice) [106]	↓ ROS production (urolithins: 0.01–31.25 μg/mL, in vitro, HL-60 myelomonocytic cells) [107]; (urolithin A: 2–40 μM, in vitro, LPS-stimulated RAW264 murine macrophage) [108]; (urolithin A: 10 μM, in vitro, LPS-primed BMDMs) [109]	↓ PI3-K/Akt/NF-κB and JNK/AP-1 [108]; ↓ NLRP3 inflammasome (urolithin A: 1–10 μM, in vitro, LPS-primed BMDMs; 20 or 100 mg/kg/day, 3 days, in vivo, mice with MSU-induced peritonitis) [109]	AMPK/SIRT1 activation [110] ^d^	Systemic exposure: urolithins and their phase-2 enzyme conjugates (methylated sulfated, and glucuronidated forms) achieve physiologically relevant concentrations through enterohepatic recirculation [112] CNS exposure: in silico predictions of urolithins BBB permeability [112]; reported detection through mass spectroscopic methods of urolithin B in brain tissues of rats after intravenous delivery [112]
Indole derivatives ^f^	Tryptophan-derived metabolite (with tryptophan being abundant in soy products, legumes, seeds, and fermented foods)	promotion of microbial community diversity via AhR/IL-22 signaling (IAld: 18 mg/kg, in vivo, C57BL/6 mice *C. albicans*-infected or DSS-induced colitis) [114]	↑ tight junctions (indole derivatives: 20 mg/kg/day, 4 weeks, in vivo, APP/PS1 transgenic mice) [113]; PXR (IPA: EC_50_ = 120 μM on hPXR and 0.55 μM on mPXR, in vitro, 293T cells; 10, 20, and 40 mg/kg/day, 4 days, in vivo, *Nr1i2^+/+^* and *Nr1i2^–/–^* transgenic mice) [116]	hydroxyl radical scavenging (IPA: 0–150 μM, in vitro, cell-free chemical assay) [118]; protection against oxidative tissue damage (IPA: 1–50 μM, in vitro, E-18 fetal rat primary hippocampal neurons and SK-N-SH human neuroblastoma cells, PC12 cells) [118]; ↓ lipid peroxidation (IPA: 10 mg/kg/day, 15 days, ex vivo, hippocampal CA1) [119]	AhR-mediated anti-inflammatory activity [multiple studies; doses (10–500 μM IAA) depending on the models; see the cited reference for details] [117]; ↓ NF-κB activation and COX-2 expression, ↓ NLRP3 inflammasome (indole derivatives: 20 mg/kg/day, 4 weeks, in vivo, APP/PS1 transgenic mice) [113]; ↑IFN-I/AhR signaling axis (IPA and IAld: 20 mg/kg/day, 2 weeks, in vivo, C57BL/6 mice with induced EAE) [120]; ↓ PXR-dependent NF-κB and BACE1 signaling [IPA: 20 mg/kg/day, 2 weeks, in vivo, male 5×FAD-transgenic mice with PXR knockdown (AAV-hSyn-PXR-shRNA) in the hippocampus] [121]	↑ BNIP3L-dependent mitophagy (IPA: 40 mg/kg/day, 2 weeks, in vivo, male C57BL/6J mice) [123]	IPA identified in the plasma and cerebrospinal fluid of human [118]. Indole derivatives detected in human serum samples through metabolomic analyses and direct measurements in urine samples [120]. IPA supplementation APP/PS1 mice markedly increased serum IPA levels [121]. Serum IPA levels were significantly lower in human volunteers, including patients with AD, compared to healthy controls [121]. CNS exposure: indirect effects of indole derivatives after oral administration in male Mongolian gerbils [119]. Peripherally administered indoxyl-3-sulfate crosses the BBB and activates AhR in astrocytes [120]. IPA was detected in the brain of 5×FAD transgenic mice within 1 h after injection [121].

^a^ Where relevant, the tested doses and the experimental models used are indicated in parentheses; ^b^ Data are referred to gut barrier integrity; data on BBB integrity are reported in blue; ^c^ dietary natural compounds; ^d^ doses/concentrations and experimental models are not reported in the table when the cited reference is a review article describing multiple studies. Readers are referred to the cited references; ^e^ human studies are included; ^f^ microbial metabolites; ^g^ PCA is also naturally present in several plant-derived foods, including berries, red wine, fruits, and vegetables; ^h^ no direct evidence of antibacterial activity against intestinal pathogens has been reported to date. Anti-adhesive activity has been demonstrated against uropathogenic *E. coli*, but this evidence does not directly support an antibacterial effect on gut pathogens [125]; ^i^ no direct evidence linking PVLs to gut barrier integrity has been reported to date. Dobani et al. (2025) discuss PVLs among flavan-3-ol-derived metabolites in relation to gut barrier integrity, but the reported evidence is not specific to PVLs [126].

## Data Availability

No new data were created or analyzed in this study. Data sharing is not applicable to this article.

## Abbreviations

The following abbreviations are used in this manuscript:

3,4-DHPA or DOPAC	3,4-Dihydroxyphenylacetic acid
ACTH	Adrenocorticotropic hormone
AD	Alzheimer’s disease
ADMET	Absorption–Distribution–Metabolism–Excretion–Toxicity
AhR	Aryl hydrocarbon receptor
Akt	Protein kinase B
ALS	Amyotrophic lateral sclerosis
AMPK	AMP-activated protein kinase
AP-1	Activator protein-1
APP	Amyloid precursor protein
AREs	Antioxidant response elements
Aβ	Amyloid-β
BACE1	β-Site APP-cleaving enzyme 1
BBB	Blood–brain barrier
BDNF	Brain-derived neurotrophic factor
CAT	Catalase
CatB	Cathepsin B
CNS	Central nervous system
COX-2	Cyclooxygenase-2
CRF	Corticotropin-releasing factor
CRH	Hypothalamic corticotropin-releasing hormone
CSF1	Colony-stimulating factor 1
DHCA	Dihydrocaffeic acid
FcR	Fc receptor
FFAR2	Free fatty acid receptor 2, also reported as GPR43
FFAR3	Free fatty acid receptor 3, also reported as GPR41
FGF	Fibroblast growth factor
GDNF	Glial cell line-derived neurotrophic factor
GPCRs	G protein-coupled receptors
GPR41	G protein-coupled receptor 41, also reported as FFAR3
GPR43	G protein-coupled receptor 43, also reported as FFAR2
GPx	Glutathione peroxidase
GR	Glutathione reductase
HD	Huntington’s disease
HDAC	Histone deacetylase
hMCTs	Human monocarboxylate transporters
HO-1	Heme oxygenase-1
HPA	Hypothalamic–pituitary–adrenal
IAA	Indole-3-acetic acid
IAld	Indole-3-aldehyde
Iba-1	Ionized calcium-binding adapter molecule 1
IFN-I	Type I interferon
IFNγ	Interferon-γ
IGF-1	Insulin-like growth factor 1
IL-10	Interleukin-10
IL-13	Interleukin-13
IL-18	Interleukin-18
IL-1β	Interleukin-1β
IL-22	Interleukin-22
IL-4	Interleukin-4
IL-6	Interleukin-6
IL-8	Interleukin-8
ILA	Indole-3-lactic acid
iNOS	Inducible nitric oxide synthase
IPA	Indole-3-propionic acid
IκB	Inhibitor of κB
JNK	c-Jun N-terminal kinase
Keap1	Kelch-like ECH-associating protein 1
LPS	Lipopolysaccharide
LXR	Liver X receptor
MAPK	Mitogen-activated protein kinase
Mfn2	Mitofusin-2
MGBA	Microbiota–Gut–Brain axis
MHC II	Major histocompatibility complex class II
mTOR	Mechanistic target of rapamycin
MUC2	Mucin 2
MyD88	Myeloid differentiation primary response 88
NAD^+^	Nicotinamide adenine dinucleotide
NADPH	Nicotinamide adenine dinucleotide phosphate
NDs	Neurodegenerative diseases
NF-κB	Nuclear factor kappa-light-chain-enhancer of activated B cells
NGF	Nerve growth factor
NLRP3	NOD-like receptor pyrin domain containing 3
NMDA	*N*-methyl-d-aspartate
NO	Nitric oxide
NOD	Nucleotide-binding oligomerization domain
NOX	NADPH oxidase
NQO-1	NAD(P)H quinone dehydrogenase 1
Nrf2	Nuclear factor erythroid 2-related factor 2
NT-4/5	Neurotrophin-4/5
PC12	Pheochromocytoma
PCA	Protocatechuic acid
PD	Parkinson’s disease
PGC-1α	PPAR-γ coactivator 1α
PGE2	Prostaglandin E2
PI3K	Phosphoinositide 3-kinase
PPARγ	Peroxisome proliferator-activated receptor-γ
PS1	Presenilin 1
PSD-95	Postsynaptic density protein 95
PVLs	Phenyl-γ-valerolactones
PXR	Pregnane X receptor
RNS	Reactive nitrogen species
ROS	Reactive oxygen species
RXR	Retinoid X receptor
SAR	Structure Activity Relationships
SCFAs	Short-chain fatty acids
SIRT1	Sirtuin 1
SOD	Superoxide dismutase
TGF-β	Transforming growth factor-β
ThPOK	T-helper-inducing POZ/Krüppel-like factor
ThT	Thioflavin T
TLR	Toll-like receptor
TLR4	Toll-like receptor 4
*TNF*	Gene encoding TNF-α
TNF-α	Tumor necrosis factor-α
Trk	Tropomyosin receptor kinase
TXNIP	Thioredoxin-interacting protein
ZO-1	Zonula occludens-1
α-Syn	α-Synuclein

## References

[1] WHO (2026). Dementia. https://www.who.int/news-room/fact-sheets/detail/dementia.

[2] Scarian E., Viola C., Dragoni F., Di Gerlando R., Rizzo B., Diamanti L., Gagliardi S., Bordoni M., Pansarasa O. (2024). New Insights into Oxidative Stress and Inflammatory Response in Neurodegenerative Diseases. Int. J. Mol. Sci..

[3] Peggion C., Calì T., Brini M. (2024). Mitochondria Dysfunction and Neuroinflammation in Neurodegeneration: Who Comes First?. Antioxidants.

[4] Gu Y., Zhao X., Zhang N., Yang Y., Yi Y., Shao Q., Liu M., Zhang X. (2024). Mitochondrial Dysfunction as a Therapeutic Strategy for Neurodegenerative Diseases: Current Insights and Future Directions. Ageing Res. Rev..

[5] Karvandi M.S., Sheikhzadeh Hesari F., Aref A.R., Mahdavi M. (2023). The Neuroprotective Effects of Targeting Key Factors of Neuronal Cell Death in Neurodegenerative Diseases: The Role of ER Stress, Oxidative Stress, and Neuroinflammation. Front. Cell. Neurosci..

[6] Manful C.F., Fordjour E., Subramaniam D., Sey A.A., Abbey L., Thomas R. (2025). Antioxidants and Reactive Oxygen Species: Shaping Human Health and Disease Outcomes. Int. J. Mol. Sci..

[7] Singh A., Kukreti R., Saso L., Kukreti S. (2019). Oxidative Stress: A Key Modulator in Neurodegenerative Diseases. Molecules.

[8] Selvaraj N.R., Nandan D., Nair B.G., Nair V.A., Venugopal P., Aradhya R. (2025). Oxidative Stress and Redox Imbalance: Common Mechanisms in Cancer Stem Cells and Neurodegenerative Diseases. Cells.

[9] Santos D.F., Simão S., Nóbrega C., Bragança J., Castelo-Branco P., Araújo I.M., Consortium A.S. (2024). Oxidative Stress and Aging: Synergies for Age Related Diseases. FEBS Lett..

[10] Solleiro-Villavicencio H., Rivas-Arancibia S. (2018). Effect of Chronic Oxidative Stress on Neuroinflammatory Response Mediated by CD4^+^T Cells in Neurodegenerative Diseases. Front. Cell. Neurosci..

[11] Chen L., Deng H., Cui H., Fang J., Zuo Z., Deng J., Li Y., Wang X., Zhao L. (2018). Inflammatory Responses and Inflammation-Associated Diseases in Organs. Oncotarget.

[12] Sequeira L., Benfeito S., Fernandes C., Lima I., Peixoto J., Alves C., Machado C.S., Gaspar A., Borges F., Chavarria D. (2024). Drug Development for Alzheimer’s and Parkinson’s Disease: Where Do We Go Now?. Pharmaceutics.

[13] Cryan J.F., O’Riordan K.J., Cowan C.S.M., Sandhu K.V., Bastiaanssen T.F.S., Boehme M., Codagnone M.G., Cussotto S., Fulling C., Golubeva A.V. (2019). The Microbiota-Gut-Brain Axis. Physiol. Rev..

[14] Tarawneh R., Penhos E. (2022). The Gut Microbiome and Alzheimer’s Disease: Complex and Bidirectional Interactions. Neurosci. Biobehav. Rev..

[15] Chen S.-J., Lin C.-H. (2022). Gut Microenvironmental Changes as a Potential Trigger in Parkinson’s Disease through the Gut-Brain Axis. J. Biomed. Sci..

[16] Padhi P., Worth C., Zenitsky G., Jin H., Sambamurti K., Anantharam V., Kanthasamy A., Kanthasamy A.G. (2022). Mechanistic Insights Into Gut Microbiome Dysbiosis-Mediated Neuroimmune Dysregulation and Protein Misfolding and Clearance in the Pathogenesis of Chronic Neurodegenerative Disorders. Front. Neurosci..

[17] Loh J.S., Mak W.Q., Tan L.K.S., Ng C.X., Chan H.H., Yeow S.H., Foo J.B., Ong Y.S., How C.W., Khaw K.Y. (2024). Microbiota–Gut–Brain Axis and Its Therapeutic Applications in Neurodegenerative Diseases. Signal Transduct. Target Ther..

[18] Kowalski K., Mulak A. (2019). Brain-Gut-Microbiota Axis in Alzheimer’s Disease. J. Neurogastroenterol. Motil..

[19] Chen N., Yao P., Farid M.S., Zhang T., Luo Y., Zhao C. (2025). Effect of Bioactive Compounds in Processed *Camellia sinensis* Tea on the Intestinal Barrier. Food Res. Int..

[20] Mohd Sairazi N.S., Sirajudeen K. (2020). Natural Products and Their Bioactive Compounds: Neuroprotective Potentials against Neurodegenerative Diseases. Evid. Based Complement. Altern. Med..

[21] Toschi A., Piva A., Grilli E. (2022). Phenol-Rich Botanicals Modulate Oxidative Stress and Epithelial Integrity in Intestinal Epithelial Cells. Animals.

[22] Marino M., Venturi S., Rendine M., Porrini M., Gardana C., Klimis-Zacas D., Bo’ C.D., Riso P. (2023). Wild Blueberry (*V. angustifolium*) Improves TNFα-Induced Cell Barrier Permeability through Claudin-1 and Oxidative Stress Modulation in Caco-2 Cells. Food Funct..

[23] Obrenovich M.E.M. (2018). Leaky Gut, Leaky Brain?. Microorganisms.

[24] Sandoval-Ramírez B.A., Catalán Ú., Pedret A., Valls R.M., Motilva M.J., Rubió L., Solà R. (2021). Exploring the Effects of Phenolic Compounds to Reduce Intestinal Damage and Improve the Intestinal Barrier Integrity: A Systematic Review of in Vivo Animal Studies. Clin. Nutr..

[25] Zafari R., Goudarzi N., Kamroo A., Tafti M.F., Ghorbani A., Talebian N., Najafi S., Shahbazian S., Rahmanian M., Parvardeh S. (2025). The Effects of Polyphenols on Gut Microbial Metabolites and Composition in Neurodegenerative Diseases: A Systematic Review. Nutr. Metab..

[26] Salinas E., Reyes-Pavón D., Cortes-Perez N.G., Torres-Maravilla E., Bitzer-Quintero O.K., Langella P., Bermúdez-Humarán L.G. (2021). Bioactive Compounds in Food as a Current Therapeutic Approach to Maintain a Healthy Intestinal Epithelium. Microorganisms.

[27] Aburto M.R., Cryan J.F. (2024). Gastrointestinal and Brain Barriers: Unlocking Gates of Communication across the Microbiota–Gut–Brain Axis. Nat. Rev. Gastroenterol. Hepatol..

[28] Parker A., Fonseca S., Carding S.R. (2019). Gut Microbes and Metabolites as Modulators of Blood-Brain Barrier Integrity and Brain Health. Gut Microbes.

[29] Cobley J.N., Fiorello M.L., Bailey D.M. (2018). 13 Reasons Why the Brain Is Susceptible to Oxidative Stress. Redox Biol..

[30] Zhao Y., Zhao B. (2013). Oxidative Stress and the Pathogenesis of Alzheimer’s Disease. Oxid. Med. Cell Longev..

[31] Kensler T.W., Wakabayashi N., Biswal S. (2007). Cell Survival Responses to Environmental Stresses Via the Keap1-Nrf2-ARE Pathway. Annu. Rev. Pharmacol. Toxicol..

[32] Wardyn J.D., Ponsford A.H., Sanderson C.M. (2015). Dissecting Molecular Cross-Talk between Nrf2 and NF-κB Response Pathways. Biochem. Soc. Trans..

[33] Di Vincenzo F., Del Gaudio A., Petito V., Lopetuso L.R., Scaldaferri F. (2024). Gut Microbiota, Intestinal Permeability, and Systemic Inflammation: A Narrative Review. Intern. Emerg. Med..

[34] Park B.S., Lee J.-O. (2013). Recognition of Lipopolysaccharide Pattern by TLR4 Complexes. Exp. Mol. Med..

[35] Sun Y., Koyama Y., Shimada S. (2022). Inflammation From Peripheral Organs to the Brain: How Does Systemic Inflammation Cause Neuroinflammation?. Front. Aging Neurosci..

[36] Kielian T. (2006). Toll-like Receptors in Central Nervous System Glial Inflammation and Homeostasis. J. Neurosci. Res..

[37] Wu L., Xian X., Xu G., Tan Z., Dong F., Zhang M., Zhang F. (2022). Toll-Like Receptor 4: A Promising Therapeutic Target for Alzheimer’s Disease. Mediat. Inflamm..

[38] Rong S., Yang C., Wang F., Wu Y., Sun K., Sun T., Wu Z. (2022). Amentoflavone Exerts Anti-Neuroinflammatory Effects by Inhibiting TLR4/MyD88/NF-κB and Activating Nrf2/HO-1 Pathway in Lipopolysaccharide-Induced BV2 Microglia. Mediat. Inflamm..

[39] Swanson K.V., Deng M., Ting J.P.-Y. (2019). The NLRP3 Inflammasome: Molecular Activation and Regulation to Therapeutics. Nat. Rev. Immunol..

[40] Franchi L., Eigenbrod T., Muñoz-Planillo R., Nuñez G. (2009). The Inflammasome: A Caspase-1-Activation Platform That Regulates Immune Responses and Disease Pathogenesis. Nat. Immunol..

[41] Jin X., Liu M.-Y., Zhang D.-F., Zhong X., Du K., Qian P., Gao H., Wei M.-J. (2019). Natural Products as a Potential Modulator of Microglial Polarization in Neurodegenerative Diseases. Pharmacol. Res..

[42] Peng M., Li N., Wang H., Li Y., Liu H., Luo Y., Lang B., Zhang W., Li S., Tian L. (2025). Macrophages: Subtypes, Distribution, Polarization, Immunomodulatory Functions, and Therapeutics. MedComm.

[43] Yan L., Wang J., Cai X., Liou Y., Shen H., Hao J., Huang C., Luo G., He W. (2024). Macrophage Plasticity: Signaling Pathways, Tissue Repair, and Regeneration. MedComm.

[44] Colonna M., Butovsky O. (2017). Microglia Function in the Central Nervous System During Health and Neurodegeneration. Annu. Rev. Immunol..

[45] Loboda A., Damulewicz M., Pyza E., Jozkowicz A., Dulak J. (2016). Role of Nrf2/HO-1 System in Development, Oxidative Stress Response and Diseases: An Evolutionarily Conserved Mechanism. Cell. Mol. Life Sci..

[46] Jurga A.M., Paleczna M., Kuter K.Z. (2020). Overview of General and Discriminating Markers of Differential Microglia Phenotypes. Front. Cell. Neurosci..

[47] Cantó C., Gerhart-Hines Z., Feige J.N., Lagouge M., Noriega L., Milne J.C., Elliott P.J., Puigserver P., Auwerx J. (2009). AMPK Regulates Energy Expenditure by Modulating NAD^+^ Metabolism and SIRT1 Activity. Nature.

[48] Chen J., Liu B., Yao X., Yang X., Sun J., Yi J., Xue F., Zhang J., Shen Y., Chen B. (2025). AMPK/SIRT1/PGC-1α Signaling Pathway: Molecular Mechanisms and Targeted Strategies From Energy Homeostasis Regulation to Disease Therapy. CNS Neurosci. Ther..

[49] Breit S., Kupferberg A., Rogler G., Hasler G. (2018). Vagus Nerve as Modulator of the Brain–Gut Axis in Psychiatric and Inflammatory Disorders. Front. Psychiatry.

[50] Mueller-Steiner S., Zhou Y., Arai H., Roberson E.D., Sun B., Chen J., Wang X., Yu G., Esposito L., Mucke L. (2006). Antiamyloidogenic and Neuroprotective Functions of Cathepsin B: Implications for Alzheimer’s Disease. Neuron.

[51] Feng T., Tammineni P., Agrawal C., Jeong Y.Y., Cai Q. (2017). Autophagy-Mediated Regulation of BACE1 Protein Trafficking and Degradation. J. Biol. Chem..

[52] Giorgi C., Bouhamida E., Danese A., Previati M., Pinton P., Patergnani S. (2021). Relevance of Autophagy and Mitophagy Dynamics and Markers in Neurodegenerative Diseases. Biomedicines.

[53] Wang Q., Xue H., Yue Y., Hao S., Huang S.-H., Zhang Z. (2022). Role of Mitophagy in the Neurodegenerative Diseases and Its Pharmacological Advances: A Review. Front. Mol. Neurosci..

[54] Lu J., Di Florio D.N., Boya P., Maday S., Springer W., Chu C.T. (2026). Autophagy and Mitophagy at the Synapse and beyond: Implications for Learning, Memory and Neurological Disorders. Autophagy.

[55] Santhiravel S., Bekhit A.E.-D.A., Mendis E., Jacobs J.L., Dunshea F.R., Rajapakse N., Ponnampalam E.N. (2022). The Impact of Plant Phytochemicals on the Gut Microbiota of Humans for a Balanced Life. Int. J. Mol. Sci..

[56] Arias-Sánchez R.A., Torner L., Fenton Navarro B. (2023). Polyphenols and Neurodegenerative Diseases: Potential Effects and Mechanisms of Neuroprotection. Molecules.

[57] Jalouli M., Rahman M.A., Biswas P., Rahman H., Harrath A.H., Lee I.-S., Kang S., Choi J., Park M.N., Kim B. (2025). Targeting Natural Antioxidant Polyphenols to Protect Neuroinflammation and Neurodegenerative Diseases: A Comprehensive Review. Front. Pharmacol..

[58] Cheng H., Zhang D., Wu J., Liu J., Zhou Y., Tan Y., Feng W., Peng C. (2023). Interactions between Gut Microbiota and Polyphenols: A Mechanistic and Metabolomic Review. Phytomedicine.

[59] Wan M.L.Y., Co V.A., El-Nezami H. (2020). Dietary Polyphenol Impact on Gut Health and Microbiota. Crit. Rev. Food Sci. Nutr..

[60] Alves-Santos A.M., Sugizaki C.S.A., Lima G.C., Naves M.M.V. (2020). Prebiotic Effect of Dietary Polyphenols: A Systematic Review. J. Funct. Foods.

[61] Verhoog S., Taneri P.E., Roa Díaz Z.M., Marques-Vidal P., Troup J.P., Bally L., Franco O.H., Glisic M., Muka T. (2019). Dietary Factors and Modulation of Bacteria Strains of *Akkermansia muciniphila* and *Faecalibacterium prausnitzii*: A Systematic Review. Nutrients.

[62] Yang Q., Liang Q., Balakrishnan B., Belobrajdic D.P., Feng Q.-J., Zhang W. (2020). Role of Dietary Nutrients in the Modulation of Gut Microbiota: A Narrative Review. Nutrients.

[63] Makarewicz M., Drożdż I., Tarko T., Duda-Chodak A. (2021). The Interactions between Polyphenols and Microorganisms, Especially Gut Microbiota. Antioxidants.

[64] Cushnie T.P.T., Lamb A.J. (2011). Recent Advances in Understanding the Antibacterial Properties of Flavonoids. Int. J. Antimicrob. Agents.

[65] Daglia M. (2012). Polyphenols as Antimicrobial Agents. Curr. Opin. Biotechnol..

[66] Yang G., Bibi S., Du M., Suzuki T., Zhu M.-J. (2016). Regulation of the Intestinal Tight Junction by Natural Polyphenols: A Mechanistic Perspective. Crit. Rev. Food Sci. Nutr..

[67] Singh A., Yau Y.F., Leung K.S., El-Nezami H., Lee J.C.-Y. (2020). Interaction of Polyphenols as Antioxidant and Anti-Inflammatory Compounds in Brain–Liver–Gut Axis. Antioxidants.

[68] Kim Y., Cho A.Y., Kim H.C., Ryu D., Jo S.A., Jung Y.-S. (2022). Effects of Natural Polyphenols on Oxidative Stress-Mediated Blood-Brain Barrier Dysfunction. Antioxidants.

[69] Wang H.C., Brumaghim J.L. (2011). Polyphenol Compounds as Antioxidants for Disease Prevention: Reactive Oxygen Species Scavenging, Enzyme Regulation, and Metal Chelation Mechanisms in *E. coli* and Human Cells. ACS Symp. Ser..

[70] Azam S., Jakaria M., Kim I.-S., Kim J., Haque M.E., Choi D.-K. (2019). Regulation of Toll-Like Receptor (TLR) Signaling Pathway by Polyphenols in the Treatment of Age-Linked Neurodegenerative Diseases: Focus on TLR4 Signaling. Front. Immunol..

[71] Scapagnini G., Sonya V., Nader A.G., Calogero C., Zella D., Fabio G. (2011). Modulation of Nrf2/ARE Pathway by Food Polyphenols: A Nutritional Neuroprotective Strategy for Cognitive and Neurodegenerative Disorders. Mol. Neurobiol..

[72] Jasim M.H., Saadoon Abbood R., Sanghvi G., Roopashree R., Uthirapathy S., Kashyap A., Sabarivani A., Ray S., Mustafa Y.F., Yasin H.A. (2025). Flavonoids in the Regulation of Microglial-Mediated Neuroinflammation; Focus on Fisetin, Rutin, and Quercetin. Exp. Cell Res..

[73] Feng Y., Yu X., Han J. (2024). Quercetin Regulates the Polarization of Microglia through the NRF2/HO1 Pathway and Mitigates Alzheimer’s Disease. Actas Esp. Psiquiatr..

[74] Zhao M., Duan J., Shen Y., Liu Y., Zeng K. (2025). Recent Advances in Neuroinflammation Prevention and Therapy: The Role of Natural Products and Underlying Mechanisms Based on Molecular Targets. Br. J. Pharmacol..

[75] Palabiyik A.A., Palabiyik E. (2025). Pharmacological Approaches to Enhance Mitochondrial Biogenesis: Focus on PGC-1A, AMPK, and SIRT1 in Cellular Health. Mol. Biol. Rep..

[76] Dhakal S., Kushairi N., Phan C.W., Adhikari B., Sabaratnam V., Macreadie I. (2019). Dietary Polyphenols: A Multifactorial Strategy to Target Alzheimer’s Disease. Int. J. Mol. Sci..

[77] Liang Z., Currais A., Soriano-Castell D., Schubert D., Maher P. (2021). Natural Products Targeting Mitochondria: Emerging Therapeutics for Age-Associated Neurological Disorders. Pharmacol. Ther..

[78] Morrison D.J., Preston T. (2016). Formation of Short Chain Fatty Acids by the Gut Microbiota and Their Impact on Human Metabolism. Gut Microbes.

[79] Yu J., Li W., Xu Y., Tang J. (2026). Gut Microbiota-Derived Short-Chain Fatty Acids (SCFAs): Immunomodulatory Effects and Therapeutic Potential in Infections. Clin. Microbiol. Rev..

[80] Burger-van Paassen N., Vincent A., Puiman P.J., van der Sluis M., Bouma J., Boehm G., van Goudoever J.B., van Seuningen I., Renes I.B. (2009). The Regulation of Intestinal Mucin MUC2 Expression by Short-Chain Fatty Acids: Implications for Epithelial Protection. Biochem. J..

[81] Wang H.-B., Wang P.-Y., Wang X., Wan Y.-L., Liu Y.-C. (2012). Butyrate Enhances Intestinal Epithelial Barrier Function via Up-Regulation of Tight Junction Protein Claudin-1 Transcription. Dig. Dis. Sci..

[82] Caetano-Silva M.E., Rund L., Hutchinson N.T., Woods J.A., Steelman A.J., Johnson R.W. (2023). Inhibition of Inflammatory Microglia by Dietary Fiber and Short-Chain Fatty Acids. Sci. Rep..

[83] Silva Y.P., Bernardi A., Frozza R.L. (2020). The Role of Short-Chain Fatty Acids From Gut Microbiota in Gut-Brain Communication. Front. Endocrinol..

[84] Fock E., Parnova R. (2023). Mechanisms of Blood–Brain Barrier Protection by Microbiota-Derived Short-Chain Fatty Acids. Cells.

[85] Donohoe D.R., Garge N., Zhang X., Sun W., O’Connell T.M., Bunger M.K., Bultman S.J. (2011). The Microbiome and Butyrate Regulate Energy Metabolism and Autophagy in the Mammalian Colon. Cell Metab..

[86] Nazzaro F., Coppola F., Fratianni F., Abdalrazeq M., Ombra M.N., De Giulio B., Coppola R., Zengin G. (2025). Polyphenols Bioactive Metabolites, and Their Anti-Biofilm and Neuroprotective Potential. Foods.

[87] Ajiboye T.O., Habibu R.S., Saidu K., Haliru F.Z., Ajiboye H.O., Aliyu N.O., Ibitoye O.B., Uwazie J.N., Muritala H.F., Bello S.A. (2017). Involvement of Oxidative Stress in Protocatechuic Acid-mediated Bacterial Lethality. Microbiologyopen.

[88] Xia L., Lin X., Zhou Y., Li Y., Liao Y., Lin Y., Lin L., Wu P., Xie J. (2026). The Effects of Phenolic Acid Supplementation on Intestinal Barrier Function: A Review. Food Funct..

[89] Sroka Z., Cisowski W. (2003). Hydrogen Peroxide Scavenging, Antioxidant and Anti-Radical Activity of Some Phenolic Acids. Food Chem. Toxicol..

[90] An L.J., Guan S., Shi G.F., Bao Y.M., Duan Y.L., Jiang B. (2006). Protocatechuic Acid from Alpinia Oxyphylla against MPP+-Induced Neurotoxicity in PC12 Cells. Food Chem. Toxicol..

[91] Giovannini C., Scazzocchio B., Matarrese P., Varì R., D’Archivio M., Di Benedetto R., Casciani S., Dessì M.R., Straface E., Malorni W. (2008). Apoptosis Induced by Oxidized Lipids Is Associated with Up-Regulation of p66Shc in Intestinal Caco-2 Cells: Protective Effects of Phenolic Compounds. J. Nutr. Biochem..

[92] Varì R., D’Archivio M., Filesi C., Carotenuto S., Scazzocchio B., Santangelo C., Giovannini C., Masella R. (2011). Protocatechuic Acid Induces Antioxidant/Detoxifying Enzyme Expression through JNK-Mediated Nrf2 Activation in Murine Macrophages. J. Nutr. Biochem..

[93] González De Llano D., Roldán M., Parro L., Bartolomé B., Moreno-Arribas M.V. (2023). Activity of Microbial-Derived Phenolic Acids and Their Conjugates against LPS-Induced Damage in Neuroblastoma Cells and Macrophages. Metabolites.

[94] Wang H., Wang H., Wang J., Wang Q., Ma Q., Chen Y. (2015). Protocatechuic Acid Inhibits Inflammatory Responses in LPS-Stimulated BV2 Microglia via NF-κB and MAPKs Signaling Pathways. Neurochem. Res..

[95] Hornedo-Ortega R., Álvarez-Fernández M.A., Cerezo A.B., Richard T., Troncoso A.M., Garcia-Parrilla M.C. (2016). Protocatechuic Acid: Inhibition of Fibril Formation, Destabilization of Preformed Fibrils of Amyloid-β and α-Synuclein, and Neuroprotection. J. Agric. Food Chem..

[96] Li H., Zheng T., Lian F., Xu T., Yin W., Jiang Y. (2022). Anthocyanin-Rich Blueberry Extracts and Anthocyanin Metabolite Protocatechuic Acid Promote Autophagy-Lysosomal Pathway and Alleviate Neurons Damage in in Vivo and in Vitro Models of Alzheimer’s Disease. Nutrition.

[97] Wei M., Hu J., Ding Y., Wu L., Wang Q., Chen L., Zhang Q., Gu J., Zhao Z., Tan R. (2026). Protocatechuic Acid Protects Neural Stem Cells against Cerebral Ischemia-Reperfusion Injury by Suppressing JAK2/STAT3 Signaling and Restoring Mitophagy. Free Radic. Biol. Med..

[98] Angelino D., Carregosa D., Domenech-Coca C., Savi M., Figueira I., Brindani N., Jang S., Lakshman S., Molokin A., Urban J.F. (2019). 5-(Hydroxyphenyl)-γ-Valerolactone-Sulfate, a Key Microbial Metabolite of Flavan-3-Ols, Is Able to Reach the Brain: Evidence from Different in Silico, In Vitro and In Vivo Experimental Models. Nutrients.

[99] Marcolin E., Chemello C., Piovan A., Barbierato M., Morazzoni P., Ragazzi E., Zusso M. (2025). A Combination of 5-(3′,4′-Dihydroxyphenyl)-γ-Valerolactone and Curcumin Synergistically Reduces Neuroinflammation in Cortical Microglia by Targeting the NLRP3 Inflammasome and the NOX2/Nrf2 Signaling Pathway. Nutrients.

[100] Cecarini V., Cuccioloni M., Zheng Y., Bonfili L., Gong C., Angeletti M., Mena P., Del Rio D., Eleuteri A.M. (2021). Flavan-3-ol Microbial Metabolites Modulate Proteolysis in Neuronal Cells Reducing Amyloid-beta (1-42) Levels. Mol. Nutr. Food Res..

[101] Ruotolo R., Minato I., La Vitola P., Artioli L., Curti C., Franceschi V., Brindani N., Amidani D., Colombo L., Salmona M. (2020). Flavonoid-Derived Human Phenyl-γ-Valerolactone Metabolites Selectively Detoxify Amyloid-β Oligomers and Prevent Memory Impairment in a Mouse Model of Alzheimer’s Disease. Mol. Nutr. Food Res..

[102] Corral-Jara K.F., Nuthikattu S., Rutledge J., Villablanca A., Morand C., Schroeter H., Milenkovic D. (2021). Integrated Multi-Omic Analyses of the Genomic Modifications by Gut Microbiome-Derived Metabolites of Epicatechin, 5-(4′-Hydroxyphenyl)-γ-Valerolactone, in TNFalpha-Stimulated Primary Human Brain Microvascular Endothelial Cells. Front. Neurosci..

[103] García-Villalba R., Giménez-Bastida J.A., Cortés-Martín A., Ávila-Gálvez M.Á., Tomás-Barberán F.A., Selma M.V., Espín J.C., González-Sarrías A. (2022). Urolithins: A Comprehensive Update on Their Metabolism, Bioactivity, and Associated Gut Microbiota. Mol. Nutr. Food Res..

[104] Tomás-Barberán F.A., González-Sarrías A., García-Villalba R., Núñez-Sánchez M.A., Núñez-Sánchez M.A., Selma M.V., García-Conesa M.T., Espín J.C. (2016). Urolithins, the Rescue of “Old” Metabolites to Understand a “New” Concept: Metabotypes as a Nexus among Phenolic Metabolism, Microbiota Dysbiosis, and Host Health Status. Mol. Nutr. Food Res..

[105] Yasuda T., Takagi T., Asaeda K., Hashimoto H., Kajiwara M., Azuma Y., Kitae H., Hirai Y., Mizushima K., Doi T. (2024). Urolithin A-Mediated Augmentation of Intestinal Barrier Function through Elevated Secretory Mucin Synthesis. Sci. Rep..

[106] Singh R., Chandrashekharappa S., Bodduluri S.R., Baby B.V., Hegde B., Kotla N.G., Hiwale A.A., Saiyed T., Patel P., Vijay-Kumar M. (2019). Enhancement of the Gut Barrier Integrity by a Microbial Metabolite through the Nrf2 Pathway. Nat. Commun..

[107] Bialonska D., Kasimsetty S.G., Khan S.I., Ferreira D. (2009). Urolithins, Intestinal Microbial Metabolites of Pomegranate Ellagitannins, Exhibit Potent Antioxidant Activity in a Cell-Based Assay. J. Agric. Food Chem..

[108] Komatsu W., Kishi H., Yagasaki K., Ohhira S. (2018). Urolithin A Attenuates Pro-Inflammatory Mediator Production by Suppressing PI3-K/Akt/NF-κB and JNK/AP-1 Signaling Pathways in Lipopolysaccharide-Stimulated RAW264 Macrophages: Possible Involvement of NADPH Oxidase-Derived Reactive Oxygen Species. Eur. J. Pharmacol..

[109] Komatsu W., Kishi H., Uchiyama K., Ohhira S., Kobashi G. (2024). Urolithin A Suppresses NLRP3 Inflammasome Activation by Inhibiting the Generation of Reactive Oxygen Species and Prevents Monosodium Urate Crystal-Induced Peritonitis. Biosci. Biotechnol. Biochem..

[110] Jayatunga D.P.W., Hone E., Khaira H., Lunelli T., Singh H., Guillemin G.J., Fernando B., Garg M.L., Verdile G., Martins R.N. (2021). Therapeutic Potential of Mitophagy-Inducing Microflora Metabolite, Urolithin A for Alzheimer’s Disease. Nutrients.

[111] Gong Z., Huang J., Xu B., Ou Z., Zhang L., Lin X., Ye X., Kong X., Long D., Sun X. (2019). Urolithin A Attenuates Memory Impairment and Neuroinflammation in APP/PS1 Mice. J. Neuroinflammation.

[112] Yuan T., Ma H., Liu W., Niesen D.B., Shah N., Crews R., Rose K.N., Vattem D.A., Seeram N.P. (2015). Pomegranate’s Neuroprotective Effects against Alzheimer’s Disease Are Mediated by Urolithins, Its Ellagitannin-Gut Microbial Derived Metabolites. ACS Chem. Neurosci..

[113] Sun J., Zhang Y., Kong Y., Ye T., Yu Q., Kumaran Satyanarayanan S., Su K.-P., Liu J. (2022). Microbiota-Derived Metabolite Indoles Induced Aryl Hydrocarbon Receptor Activation and Inhibited Neuroinflammation in APP/PS1 Mice. Brain Behav. Immun..

[114] Zelante T., Iannitti R.G., Cunha C., De Luca A., Giovannini G., Pieraccini G., Zecchi R., D’Angelo C., Massi-Benedetti C., Fallarino F. (2013). Tryptophan Catabolites from Microbiota Engage Aryl Hydrocarbon Receptor and Balance Mucosal Reactivity via Interleukin-22. Immunity.

[115] Cervantes-Barragan L., Chai J.N., Tianero M.D., Di Luccia B., Ahern P.P., Merriman J., Cortez V.S., Caparon M.G., Donia M.S., Gilfillan S. (2017). *Lactobacillus reuteri* Induces Gut Intraepithelial CD4^+^CD8αα^+^ T Cells. Science.

[116] Venkatesh M., Mukherjee S., Wang H., Li H., Sun K., Benechet A.P., Qiu Z., Maher L., Redinbo M.R., Phillips R.S. (2014). Symbiotic Bacterial Metabolites Regulate Gastrointestinal Barrier Function via the Xenobiotic Sensor PXR and Toll-like Receptor 4. Immunity.

[117] Krishnan S., Ding Y., Saedi N., Choi M., Sridharan G.V., Sherr D.H., Yarmush M.L., Alaniz R.C., Jayaraman A., Lee K. (2018). Gut Microbiota-Derived Tryptophan Metabolites Modulate Inflammatory Response in Hepatocytes and Macrophages. Cell Rep..

[118] Chyan Y.-J., Poeggeler B., Omar R.A., Chain D.G., Frangione B., Ghiso J., Pappolla M.A. (1999). Potent Neuroprotective Properties against the Alzheimer β-Amyloid by an Endogenous Melatonin-Related Indole Structure, Indole-3-Propionic Acid. J. Biol. Chem..

[119] Hwang I.K., Yoo K.-Y., Li H., Park O.K., Lee C.H., Choi J.H., Jeong Y.-G., Lee Y.L., Kim Y.-M., Kwon Y.-G. (2009). Indole-3-Propionic Acid Attenuates Neuronal Damage and Oxidative Stress in the Ischemic Hippocampus. J. Neurosci. Res..

[120] Rothhammer V., Mascanfroni I.D., Bunse L., Takenaka M.C., Kenison J.E., Mayo L., Chao C.-C., Patel B., Yan R., Blain M. (2016). Type I Interferons and Microbial Metabolites of Tryptophan Modulate Astrocyte Activity and Central Nervous System Inflammation via the Aryl Hydrocarbon Receptor. Nat. Med..

[121] Li L., Jia M., Yang C., Zhao Y., Hu J., Zhao Y., Hu X., Ning F., Ding C., Li Q. (2025). Gut Microbial-Derived Indole-3-Propionate Improves Cognitive Function in Alzheimer’s Disease. Sci. Adv..

[122] Kim C.-S., Jung S., Hwang G.-S., Shin D.-M. (2023). Gut Microbiota Indole-3-Propionic Acid Mediates Neuroprotective Effect of Probiotic Consumption in Healthy Elderly: A Randomized, Double-Blind, Placebo-Controlled, Multicenter Trial and in Vitro Study. Clin. Nutr..

[123] Zhao L., Dong S., Tang Q., Liu J., Cui Q., Wang H., Zhang Y., Li P., Li D., Wang Y. (2026). Gut Microbiota-driven Indole-3-propionic Acid Protects against Sepsis-associated Encephalopathy via Inhibiting BNIP3L Ubiquitination and Restoring BNIP3L-dependent Mitophagy as a Potential Molecular Mechanism. Mol. Med..

[124] Owe-Larsson M., Drobek D., Iwaniak P., Kloc R., Urbanska E.M., Chwil M. (2025). Microbiota-Derived Tryptophan Metabolite Indole-3-Propionic Acid-Emerging Role in Neuroprotection. Molecules.

[125] Márquez Campos E., Stehle P., Simon M.-C. (2019). Microbial Metabolites of Flavan-3-Ols and Their Biological Activity. Nutrients.

[126] Dobani S., Kirsty Pourshahidi L., Ternan N.G., McDougall G.J., Pereira-Caro G., Bresciani L., Mena P., Almutairi T.M., Crozier A., Tuohy K.M. (2025). A Review on the Effects of Flavan-3-Ols, Their Metabolites, and Their Dietary Sources on Gut Barrier Integrity. Food Funct..

[127] Cavalluzzi M.M., Mangiatordi G.F., Nicolotti O., Lentini G. (2017). Ligand Efficiency Metrics in Drug Discovery: The Pros and Cons from a Practical Perspective. Expert Opin. Drug Discov..

[128] Heidor R., Festa Ortega J., De Conti A., Prates Ong T., Salvador Moreno F. (2012). Anticarcinogenic Actions of Tributyrin, A Butyric Acid Prodrug. Curr. Drug Targets.

[129] Williams L.M., Cao S. (2024). Harnessing and Delivering Microbial Metabolites as Therapeutics via Advanced Pharmaceutical Approaches. Pharmacol. Ther..

[130] Någren K. (2003). PET and Knockout Mice in Drug Discovery. Drug Discov. Today.

[131] Saha S., Rajpal D.K., Brown J.R. (2016). Human Microbial Metabolites as a Source of New Drugs. Drug Discov. Today.

[132] Dvořák Z., Sokol H., Mani S. (2020). Drug Mimicry: Promiscuous Receptors PXR and AhR, and Microbial Metabolite Interactions in the Intestine. Trends Pharmacol. Sci..

[133] Dvorak Z., Klapholz M., Burris T.P., Willing B.P., Gioiello A., Pellicciari R., Galli F., March J., O’Keefe S.J., Sartor R.B. (2020). Weak Microbial Metabolites: A Treasure Trove for Using Biomimicry to Discover and Optimize Drugs. Mol. Pharmacol..

[134] Zhou F., Feng X., Xu Z., Yan F., Song G., Tang L. (2025). Design, Synthesis and Biological Activity of 8-Hydroxy Modified Urolithin A Derivatives as Phosphodiesterase Type II (PDE2) Inhibitors. Bioorganic Med. Chem..

[135] Anastassova N., Stefanova D., Hristova-Avakumova N., Georgieva I., Kondeva-Burdina M., Rangelov M., Todorova N., Tzoneva R., Yancheva D. (2023). New Indole-3-Propionic Acid and 5-Methoxy-Indole Carboxylic Acid Derived Hydrazone Hybrids as Multifunctional Neuroprotectors. Antioxidants.

[136] Zheng X., Cai X., Hao H. (2022). Emerging Targetome and Signalome Landscape of Gut Microbial Metabolites. Cell Metab..

[137] Hao L., Li Z.-F., Qu Y.-N., Zhao F.-Y., Lu S.-Y., Li B.-Q., Zhang H.-Y., Wang H.-Q. (2026). IUPHAR Review. Gut Microbial Metabolites as Remote Regulators of Behavior and Neuropsychiatric Disease. Pharmacol. Res..

